# Protein domain architectures provide a fast, efficient and scalable alternative to sequence-based methods for comparative functional genomics

**DOI:** 10.12688/f1000research.9416.3

**Published:** 2017-06-27

**Authors:** Jasper J. Koehorst, Edoardo Saccenti, Peter J. Schaap, Vitor A. P. Martins dos Santos, Maria Suarez-Diez

**Affiliations:** 1 Laboratory of Systems and Synthetic Biology, Wageningen University and Research, Wageningen, Netherlands; 2 LifeGlimmer GmBH, Berlin, Germany

**Keywords:** Bacterial genomics, Bacterial functionome, Orthology, Horizontal gene transfer, clustering, semantic annotation

## Abstract

A functional comparative genome analysis is essential to understand the mechanisms underlying bacterial evolution and adaptation. Detection of functional orthologs using standard global sequence similarity methods faces several problems; the need for defining arbitrary acceptance thresholds for similarity and alignment length, lateral gene acquisition and the high computational cost for finding bi-directional best matches at a large scale. We investigated the use of protein domain architectures for large scale functional comparative analysis as an alternative method. The performance of both approaches was assessed through functional comparison of 446 bacterial genomes sampled at different taxonomic levels. We show that protein domain architectures provide a fast and efficient alternative to methods based on sequence similarity to identify groups of functionally equivalent proteins within and across taxonomic boundaries, and it is suitable for large scale comparative analysis. Running both methods in parallel pinpoints potential functional adaptations that may add to bacterial fitness.

## Introduction

Comparative analysis of genome sequences has been pivotal to unravel mechanisms shaping bacterial evolution like gene duplication, loss and acquisition ^1, 2^, and helped in shedding light on pathogenesis and genotype-phenotype associations ^3, 4^.

Comparative analysis relies on the identification of sets of orthologous and paralogous genes and subsequent transfer of function to the encoding proteins. Technically orthologs are defined as best bi-directional hits (BBH) obtained via pairwise sequence comparison among multiple species and thus exploits sequence similarity for functional grouping. Sequence similarity-based (SB) methods present a number of shortcomings. First, a generalized minimal alignment length and similarity cut-off need to be arbitrarily selected for all, which may hamper proper functional grouping. Second, sequence and function might differ across evolutionary scales. Protein sequences change faster than protein structure and proteins with same function but with low sequence similarity have been identified ^5, 6^. SB methods may fail to group them hampering a functional comparison. This limitation becomes even more critical when comparing either phylogenetically distant genomes or gene sequences that were acquired with horizontal gene transfer events. Recent technological advancements are resulting in thousands of organisms and billions of proteins being sequenced ^7^ which increases the need of methods able to perform comparisons at the larger scales.

**Figure 1.**
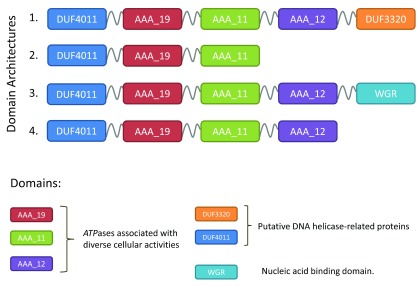
Domain architecture as a formal description of functional equivalence. Although the proteins obviously share a common core, four distinct domain architectures involving six protein domains were observed in (1) Enterobacteriaceae, (2) *H. pylori*, (3) *Pseudomonas* and (4) Cyanobacteria.

**Figure 2.**
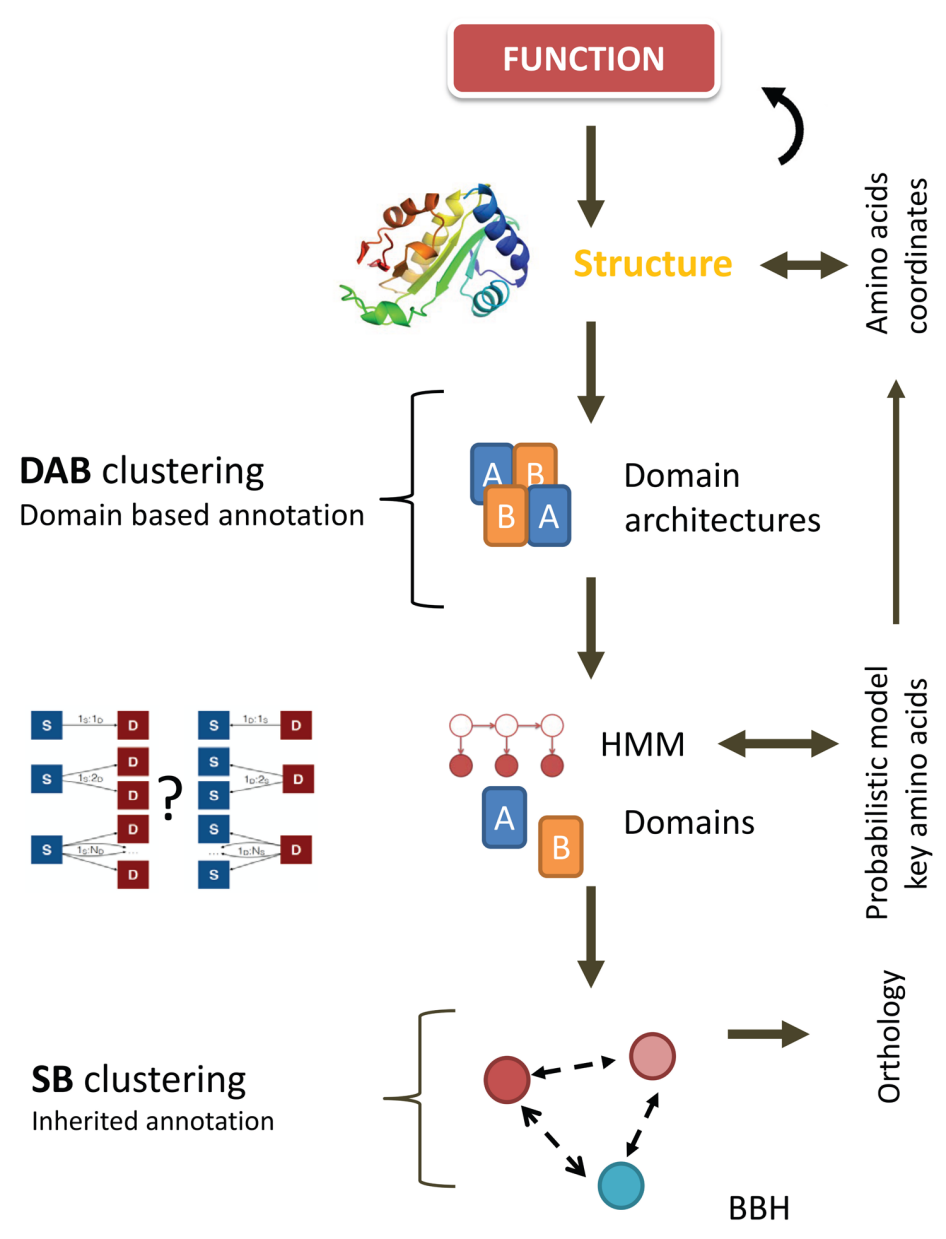
Relationship between Domain Architecture Based (DAB) and Sequence Similarity based (SB) clustering with respect to functional annotation. Domains are probabilistic models of amino acids coordinates obtained by hidden Markov modeling (HMM) built from (structure based) multiple sequence alignments. Domain architectures are linear combinations of these domains representing the functional potential of a given protein sequence and constitute the input for DAB clustering. SB-orthology clusters inherit functional annotations via best bi-directional hits above a predefined sequence similarity cut-off score.

To overcome these bottlenecks, protein domains have been suggested as an alternative for defining groups of functionally equivalent proteins ^8– 10^ and have been used to perform comparative analyses of *Escherichia coli* ^9^, *Pseudomonas* ^10^, *Streptococcus* ^11^ and for protein functional annotation ^12, 13^. A protein domain architecture describes the arrangement of domains contained in a protein and is exemplified in Figure 1. As protein domains capture key structural and functional features, protein domain architectures may be considered to be better proxies to describe functional equivalence than a global sequence similarity ^14^. The concept of using the domain architecture to precisely describe the extent of functional equivalence is exemplified in Figure 2. Moreover, once the probabilistic domain models have been defined, mining large sets of individual genome sequences for their occurrences is a considerably less demanding computational task than an exploration of all possible bi-directional hits between them ^15, 16^.

Domain architectures have been shown to be preserved at large phylogenetic distances both in prokaryotes and eukaryotes ^17, 18^. This lead to the use of protein domain architectures to classify and identify evolutionarily related proteins and to detect homologs even across evolutionarily distant species ^19– 22^. Structural information encoded in domain architectures has also been deployed to accelerate sequence search methods and to provide better homology detection. Examples are CDART ^23^ which finds homologous proteins across significant evolutionary distances using domain profiles rather than direct sequence similarity, or DeltaBlast ^24^ where a database of pre-constructed position-specific score matrix is queried before searching a protein-sequence database. Considering protein domain content, order, recurrence and position has been shown to increase the accuracy of protein function prediction ^25^ and has led to the development of tools for protein functional annotation, such as UniProt-DAAC ^26^ which uses domain architecture comparison and classification for the automatic functional annotation of large protein sets. The systematic assessment and use of domain architectures is enabled by databases containing protein domain information such as UniProt ^27^, Pfam ^28^, TIGRFAMs ^29^, InterPro ^30^, SMART ^31^ and PROSITE ^32^, that also provide graphical view of domain architectures.

Building on these observations we aim at exploring the potential of domain architecture-based (DAB) methods for large scale functional comparative analysis by comparing functionally equivalent sets of proteins, defined using domain architectures, with standard clusters of orthogonal proteins obtained with SB methods. We compared the SB and DAB approach by analysing *i)* the retrieved number of singletons ( *i.e.* clusters containing only one protein) and *ii)* the characteristics of the inferred pan- and core-genome size considering a selection of bacterial genomes (both gram positive and negative) sampled at different taxonomic levels (species, genus, family, order and phylum). We show that the DAB approach provides a fast and efficient alternative to SB methods to identify groups of functionally equivalent/related proteins for comparative genome analysis and that the functional pan-genome is more closed in comparison to the sequence based pan-genome. DAB approaches can complement standardly applied sequence similarity methods and can pinpoint potential functional adaptations.

## Methods

### Genome sequence retrieval

Bacterial species were chosen on the basis of the availability of fully sequenced genomes in the public domain: two species ( *Listeria monocytogenes* and *Helicobacter pylori*), three genera ( *Streptococcus*, *Pseudomonas*, *Bacillus*), one family (Enterobacteriaceae), one order (Corynebacteriales), and one phylum (Cyanobacteria) were selected. For each, 60 genome sequences were considered, except for *L. monocytogenes* for which only 26 complete genome sequences were available. Maximal diversity among genome sequences was ensured by sampling divergent species (when possible) at each taxonomic level. Genome sequences were retrieved from the European Nucleotide Archive database ( www.ebi.ac.uk/ena). A full list of genomes analyzed is available in the *Data availability* section.

### *De novo* genome annotation

To avoid bias due to different algorithms used for the annotation of the original deposited genome sequences, all genomes were *de novo* re-annotated using the SAPP framework (1.0.0) ^10^. In particular, the FASTA2RDF, GeneCaller (implementing Prodigal (2.6.2) ^33^) and InterPro (implementing interproscan-5.17-56.0) ^34^) modules were used to handle, re-annotate the genome sequences and store the results in the RDF data model. This resulted in 446 annotated genomes (7 × 60 genomes + 1 × 26 genomes). For each annotation step the provenance information (E-value cut off, score, originating tool or database) was stored together with annotation information in a graph database (RDF-model) and can be reproduced through the SAPP framework ( http://semantics.systemsbiology.nl).

### Retrieval of domain architecture

The positions (start and end on the protein sequence) of domains having Pfam ^28^, TIGRFAMs ^29^ and InterPro ^30^ identifiers were extracted through SPARQL querying of the graph database and domain architectures were retrieved for each protein individually. InterPro aggregates protein domain signatures from different databases. Here no pruning for redundancies has been done. Identification of domains was done using the intrinsic InterPro cut-off that represents in each case the e-values and the scoring systems of the member databases ^30^. The domain starting position was used to assess relative position in the case of overlapping domains; alphabetic ordering was used to order domains with the same starting position or when the distance between the starting position of overlapping domains was < 3 amino acids.

Labels indicating N-C terminal order of identified domains were assigned to each protein using the starting position of the domains: the same labels were assigned to proteins sharing the same domain architecture.

### Sequence similarity based clustering

To make a direct comparison possible only protein sequences containing at least one protein domain signature were considered for analysis. BBH were obtained using Blastp (2.2.28+) with an E-value cutoff of 10 ^−5^ and -max_target_seqs of 10 ^5^. OrthaGogue (1.0.3) ^35^ combined with MCL (14-137) ^36^ was used to identify protein clusters on the base of sequence similarity.

### Domain architecture based clustering

Domain architecture based clusters were built by clustering proteins with the same labels using bash terminal commands (sort, awk). The number of proteins sharing a given domain architecture in each genome was stored in a 446 × 21054 (genomes × domain architectures) matrix; from this a binarized presence-absence matrix was obtained and used solely for principal component analysis.

### Heaps’ law fitting and pan-genome openness assessment

A Heaps’ law model was fit to the abundance matrices using 5 × 10 ^3^ random genome ordering permutations and the micropan R package ^37^.

### Software

SAPP, a Semantic Annotation Pipeline with Provenance which stores results in a graph database ^10^, used for genome handling and annotation, is available at http://semantics.systemsbiology.nl. Matrix manipulations and multivariate analysis were performed using the R software (3.2.2).

## Results

SB and DAB approaches were compared by considering eight sets of genome sequences sampled at different taxonomic levels, from species to order, preserving phylogenetic diversity (see Table 1). Each set contained 60 genome sequences, except for *Listeria monocytogenes* for which only 26 complete genomes were publicly available. To facilitate the comparison between DAB and SB clusters only protein sequences that contained at least one domain were considered. On average, 85% of the protein sequences contain at least one domain from the InterPro database (see Table 1). Values range from 77±4% for Cyanobacteria to 91 ± 4% for Enterobacteriaceae (which include *E. coli*). Since the overall results were the same for gram negative and gram positive bacteria, we will show and comment only on results for the latter. Results obtained for gram negative bacteria are shown in the *Data availability section*.

**Table 1.** Comparison between DAB and SB clustering. DAB has been performed using HMM from Pfam (29.0) and InterPro (interproscan-5.17-56.0). Fraction refers to the fraction of proteins with at least one (InterPro or Pfam) protein domain. Core- and pan- indicate the sizes of the core- and pan- genomes (based on the sample) and singletons refers to the number of clusters with only one protein.

		Fraction	DAB	Pfam		DAB	InterPro		SB		
Taxon	Name	InterPro	Pfam	Core-	Pan-	Singletons	Core-	Pan-	Singletons	Core-	Pan-	Singletons
Species	*H. pylori*	0,82 ± 0,01	0,81 ± 0,01	724	1334	142	534	2888	853	1036	1503	295
Species	*L. monocytogenes*	0,89 ± 0,01	0,88 ± 0,02	1333	2142	309	1414	3415	847	2294	2937	746
Genus	*Bacillus*	0,87 ± 0,03	0,85 ± 0,03	792	5984	1474	342	16349	6745	885	9903	5505
Genus	*Pseudomonas*	0,88 ± 0,02	0,87 ± 0,02	1113	6572	1554	646	19387	7444	1453	12204	4838
Genus	*Streptococcus*	0,87 ± 0,02	0,85 ± 0,02	535	3435	845	244	8265	3276	716	4468	2116
Family	Enterobacteriaceae	0,91 ± 0,04	0,90 ± 0,05	146	6690	1664	20	19590	8173	197	10899	6715
Order	Corynebacteriales	0,83 ± 0,05	0,80 ± 0,06	475	6022	1719	130	22558	10554	605	12632	9087
Phylum	Cyanobacteria	0,77 ± 0,04	0,74 ± 0,05	400	9752	4428	120	27421	16140	511	10575	11154

### Cluster formation based on sequence similarity

**Figure 3.**
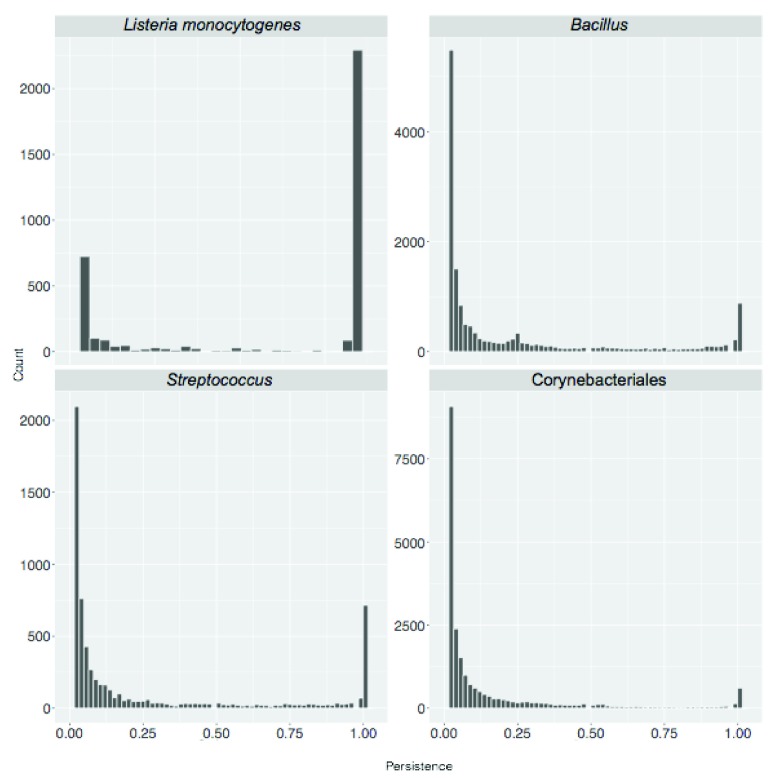
Persistence of sequence similarity based (SB) clusters. Cluster persistence is defined as the relative number of genomes with at least one protein assigned to the cluster. The frequency of SB clusters according to their persistence is shown.

A standard BBH workflow was used to obtain SB protein clusters for the eight sets. We started by calculating the total number of clusters, corresponding to the pan-genome size, as shown in Table 1. Then we considered protein cluster persistence, that is the number of genomes where at least one member of the cluster is present, divided by the total number of genomes considered. Results are shown in Figure 3.

The ratio between the size of the core-genome (clusters with persistence of 1, *i.e.* present in all genomes) and the number of singletons decreased with evolutionary distance (see Table 1). It ranged from 3.51 and 3.07 at species level ( *H. pylori* and *L. monocytogenes* respectively) to 0.05 and 0.06 when considering members of the same order (Corynebacteriales) and phylum (Cyanobacteria) respectively. A similar pattern is observed when directly comparing the sizes of the pan- and core- genomes of the sampled genomes. Within the gram negative bacteria this ratio ranges from 0.69 for members of the same species ( *H. pylori*) to 0.05 for members of the same phylum (Cyanobacteria) with intermediate values (0.12) for sequences from the same genus ( *Pseudomonas*).

### Cluster formation based on domain architectures

**Figure 4.**
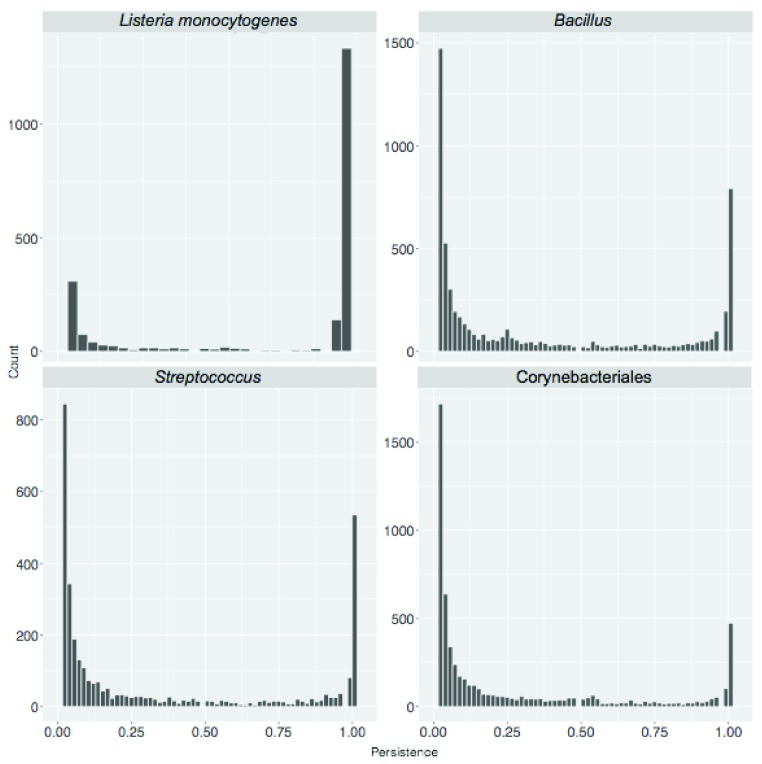
Persistence of domain architecture based (DAB) clusters. The frequency of DAB clusters according to their persistence is shown.

Domain architectures directly rely on the definition of protein domain models: those were retrieved from Pfam, InterPro and TIGRFAMs databases. However, TIGRFAMs results were not further considered because of a lower coverage. As shown in Table 1, as expected partly overlapping results were obtained when different domain databases were used. The number of singletons was larger when using InterPro rather than Pfam and for the latter we also observed larger core-genome size. These discrepancies can be due to the fact InterPro aggregates different resources (including Pfam and TIGRFAMs) and domain signatures arising from different databases are integrated with different identifiers in InterPro. In light of this we focused on results obtained using Pfam whose current release (30.0) contains hidden Markov models for over 16300 domain families. Size and persistence of groups of functionally equivalent proteins obtained using Pfam domains are presented in Figure 4.

Similar to what has been observed in the SB case we observed a decrease of the ratio between the size of the core genome and the number of singletons when higher taxonomic levels are considered. For organisms of the same species ( *H. pylori* and *L. monocytogenes*) the ratio was 5.09 and 4.30, respectively, while for member of the same order (Corynebacteriales) and phylum (Cyanobacteria) it was 0.55 and 0.009 respectively. Similarly, also the ratio between the size of the core- and pan-genome decreases as higher taxonomic levels are considered, ranging from 0.54 for *H. pylori* to 0.04 for Cyanobacteria.

### Comparison of DAB and SB clusters

**Figure 5.**
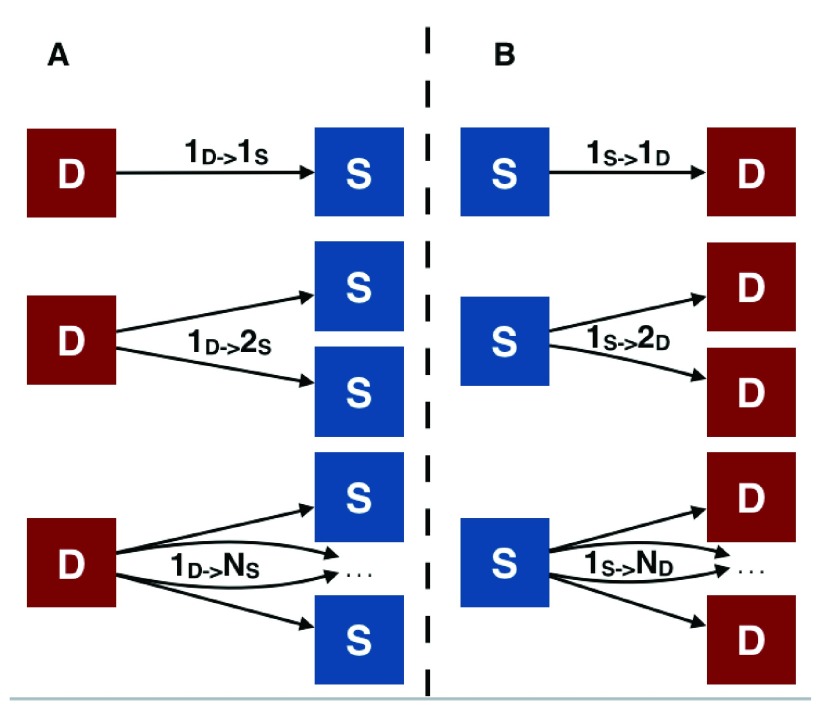
Summary of possible mismatches between DAB and SB clusters. Mismatches of SB and DAB derived clusters (marked by *S* and *D* respectively) can occur in two directions. Panel **A**: possible cases of mismatch when counting the number of SB clusters the sequences in a DAB cluster are assigned to. 1 *d* → 1 *s* denotes that all sequences from the D cluster are assigned to the same S cluster. 1 *d* → *Ns* denotes that sequences in a single D cluster are assigned to *N* distinct S clusters with *N* ≥ 1. Similarly, (panel **B**) 1 *s* → *Nd* denotes that sequences in a single S cluster are assigned to *N* distinct D clusters with *N* ≥ 1.

**Figure 6.**
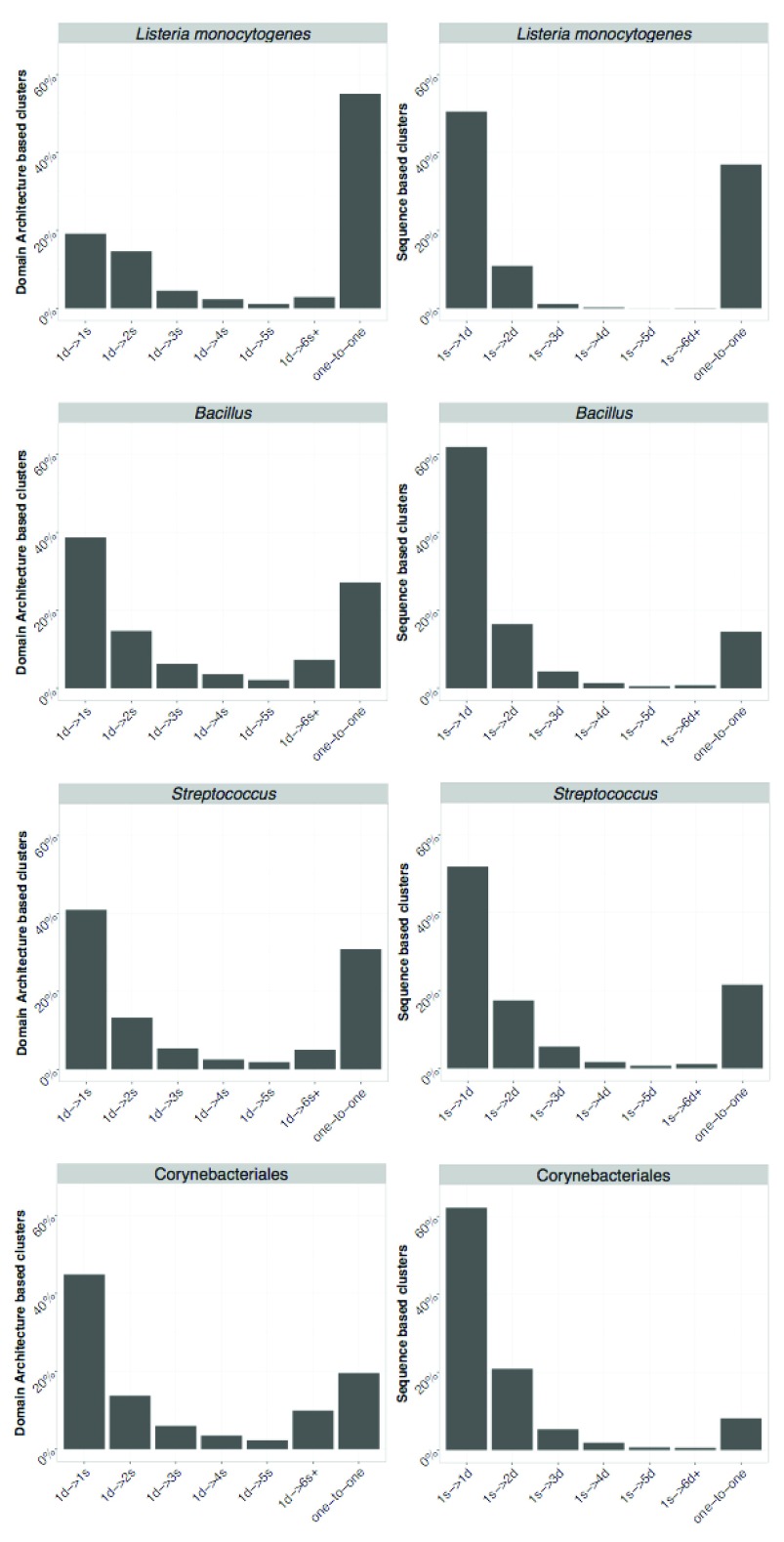
Comparison between DAB and SB clusters. On the left DAB is used as a reference and each bar represents the relative frequency of one DAB cluster containing sequences assigned to {1, 2, ... , 5} and 6 or more SB clusters and one-to-one represents the relative frequency of identical cluster. Similarly, on the right SB is used as a reference. Axis labels follow notation in Figure 5.

We compared the clusters obtained using both approaches and the proteins assigned to them. The number of one-to-one relationships (indicating a complete agreement) between SB and DAB clusters is indicated in Table 2 and ranges from 648 (for *H. pylori*) to 1680 (in *Pseudomonas*) corresponding to 50% and 25% of the pan-genome. This indicates that results of SB and DAB clustering tend to be more similar when working at closer phylogenetic distances. However, more complicated cases occur when proteins in a single SB cluster are assigned to various DAB clusters including singletons and vice versa. An overview of the possible mismatches between SB and DAB clusters is in Figure 5. The observed frequency of the different types of cluster mismatches are given in Figure 6. We observed that single domain architectures predominated the one-to-one clusters as shown in Table 3.

**Table 2.** Number of identical clusters found with SB and DAB.

Group	Clusters
*H. pylori*	648
*L. monocytogenes*	1085
*Bacillus*	1439
*Pseudomonas*	1680
*Streptococcus*	961
Enterobacteriaceae	1649
Corynebacteriales	1034
Cyanobacteria	1127

For *L. monocytogenes* we found 378 1 *d* → 1 *s* DAB cluster mismatches, ( Figure 5, panel A, top case) meaning that in those cases sequences in a DAB cluster are a subset of the sequences in the corresponding SB cluster. This lower number of sequences in the DAB cluster could be due to, for instance an insertion or expansion of a domain, leading to SB clustered sequences with partly overlapping but distinct domain architectures as is depicted in Figure 1. Similarly, there are 399 1s → 1d clusters. Each of these cases represent a sequence cluster where all the sequences share the same domain architecture, but other sequences exist with the same architecture that have not been included in the cluster due to a too low similarity score. The low similarity between sequences with the same domain architecture could be due to a horizontal acquisition of the gene or to a fast protein evolution at the sequence level. Genes acquired from high phylogenetic distances could greatly vary in sequence while presenting the same domain architecture.

**Table 3.** Composition in terms of domains (#domains) of domain architectures found within identical (one-to-one) SB and DAB clusters.

#Domains	*H. pylori*	*L. monocytogenes*	*Bacillus*	*Pseudomonas*	*Streptococcus*	Enterobacteriaceae	Cyanobacteria	Corynebacteriales
1	463	768	1119	1185	734	1312	867	772
2	133	207	229	333	164	246	182	192
3	40	76	65	107	43	64	57	45
4	8	23	18	37	13	15	14	16
5	3	9	3	10	5	6	4	5
6	0	2	2	5	1	3	3	4
7	1	0	1	3	1	3	0	0
8	0	0	1	0	0	0	0	0
9	0	0	1	0	0	0	0	0

Proteins contained in a single DAB cluster but assigned to multiple SB clusters contain mostly ABC transporters-like (PF00005) or Major Facilitator Superfamily (MFS, PF07690) domains. This is not surprising considering that such generic functions are usually associated with a high sequence diversity. Conversely, ABC transporters are found in multiple DAB clusters. However, many of them are grouped into a single SB cluster with ATPase domain containing proteins (1 *s* → *Nd* case).

We observed distinct architectures with one of two very similar domains, the GDSL-like Lipase/Acylhydrolase and the GDSL-like Lipase/Acylhydrolase *family* domain (PF00657 and PF13472 respectively) and those architectures were often seen clustered using a SB approach. However, architectures containing both domains were also identified, pointing to a degree of functional difference as a result of convergent or divergent evolution. Still, the corresponding sequences remain similar enough as to be indistinguishable when a SB approach is used. For SB clustering we also observed the case of identical protein sequences not clustered together, probably because of the tie breaking implementation when BBH are scored.

In all cases we found the size of both the pan- and the core-genome to be larger when a SB approach is used to identify gene clusters and SB approaches lead to a larger number of singletons than DAB ones. This indicates that DAB clusters are assigned to several SB clusters, many of them consisting of just one protein.

When going from species to phylum level, the ratio between the number of DAB and SB singletons changes from 0.48 and 0.41 (for *H. pylori* and *L. monocytogenes* respectively) to 0.19 and 0.40 when considering organisms of a higher taxonomic level (Corynebacteriales and Cyanobacteria respectively).

We investigated the predicted size of the pan-genome upon addition of new sequences. Heaps’ law regression can be used to estimate whether the pan-genome is open or closed ^38^ through the fitting of the decay parameter *α*; *α* < 1 indicates openness of the pan-genome (indicating that possibly many clusters remain to be identified within the considered set of sequences), while *α* > 1 indicates a closed one; the *α* values are given in Table 4. In all cases the pan-genome is predicted to be open; however, *α* values obtained using DAB clusters ( *α*_*DAB*_) are systematically closer to one than the *α*_*SB*_ obtained with the standard sequence similarity approach.

**Figure 7.**
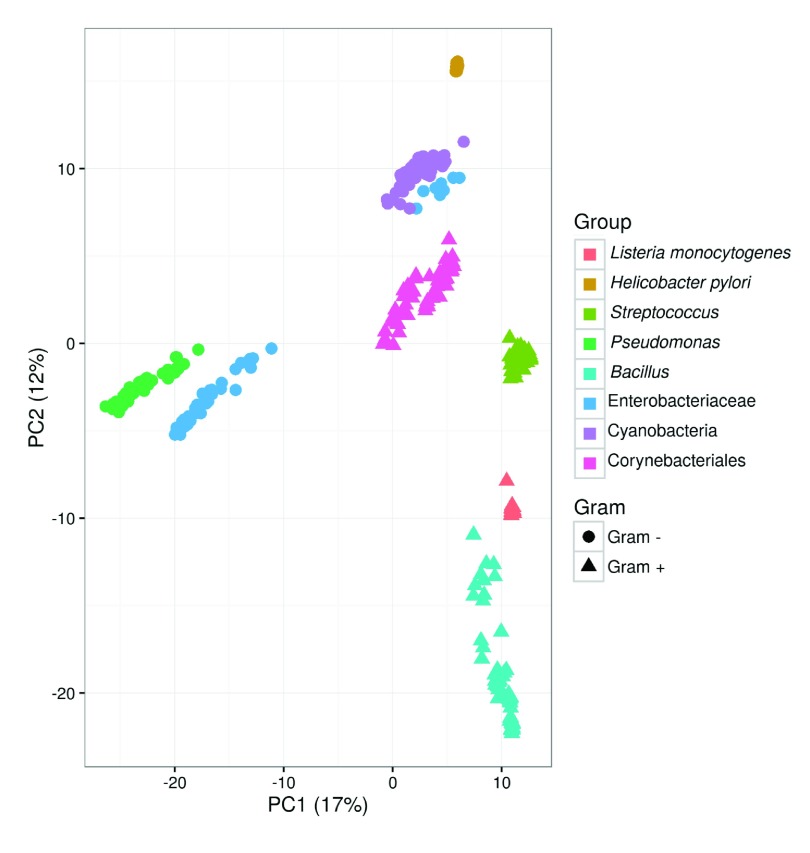
Large scale functional comparison of species. Principal component analysis of functional similarities of 446 genomes based on the presence/absence of domain architectures on the corresponding genomes. The variance explained by the first two components is indicated on axes labels.

The *α _DAB_* value retrieved for *L. monocytogenes* is strikingly low. Heaps law regression relies on the selected genomes providing a uniform sampling of selected taxon, here species. Analysis of the domain content of the selected genomes shows a divergent behaviour of strain LA111 (genome id GCA_000382925-1). This behaviour is clear in Figure 7, where GCA_000382925-1 appears as an outlier of the *L. monocytogenes* group. Removal of these outlier leads to *α _DAB_* = 1.04 and *α _SB_* = 0.64, which emphasizes the need for uniform sampling prior to Heaps regression analysis.

**Table 4.** Decay parameter ***α*** of the Heaps regression model using DAB and SB clustering.

	*α* *_DAB_*	*α* *_SB_*
*H. pylori*	0.95	0.42
*L. monocytogenes*	0.77 (1.04 *)	0.50 (0.64 *)
*Bacillus*	0.93	0.59
*Pseudomonas*	0.94	0.61
*Streptococcus*	0.87	0.72
Enterobacteriaceae	0.99	0.74
Cyanobacteria	0.64	0.58
Corynebacteriales	0.88	0.52

*α* < 1 indicates an open pan-genome.

*Values obtained upon removal of sequence GCA_000382925-1

### DAB comparison across multiple taxa

DAB clusters can be labelled by their domain architecture and since this is a formal description of functional equivalence, results of independently obtained analyses can be combined. Figure 7 shows the results of a principal component analysis of the combined DAB clusters for selected genomes from eight taxa. The first two components account for a relatively low explained variance (29%) still grouping of genomes from the same taxa is apparent. High functional similarity among genomes of the same species ( *H. pylori* and *L. monocytogenes*) is reflected by the compact clustering, while phylogenetically more distant genomes appear scattered in the functional space defined by the principal components.

## Discussion

We have shown that domain architecture-based methods can be used as an effective approach to identify clusters of functionally equivalent proteins, leading to results similar to those obtained by classical methods based on sequence similarity.

To assess whether DAB results were consistent with those of SB methods we chosen OrthaGogue as a representative of the latter class. Several tools such as COGNITOR ^39^ and MultiPARANOID ^40^ are available that implement different algorithm solutions to identify homologous sequences; however, despite different implementations, they all rely on sequence similarity as a proxy for functional equivalence. Here we considered SB methods as a golden standard for functional comparative genomics, especially when organisms within close evolutionary proximity are considered. Our aim was to investigate whether using domain architectures instead of sequence similarity alone would yield similar results, thereby justifying their use for large scale functional genome comparisons. Regarding domain architectures, we have explored different alternatives, as we have seen that the chosen database or set of reference domains plays a critical role; for example, the low coverage of TIGRFAM prevents the obtaining of reasonable clusters. The DAB approach takes advantage of the large computational effort that has already been devoted to the identification and definition of protein domains in dedicated databases such as Pfam. Protein domain models are built using large scale sequence comparisons which is an extremely computationally intensive task. However, once the domain models are defined, mining a sequence for domain occurrences is much less demanding task. Indeed, the task with the higher computational load (the definition of the domains) is performed only once and results can be stored and re-used for further analysis. This provides an effective scalable approach for large scale functional comparisons which by and large is independent of phylogenetic distances between species.

The chosen set of domain models and the database used as a reference greatly impact the results. InterPro aggregates protein domain signatures from different databases, which leads to redundancy of the domain models. This redundancy causes overlaps between the entries and an increase of the granularity of the clusters retrieved: this can bias downwards the size of the pan-genome and upwards the size of the core- genome, as shown in Table 1. In InterPro this redundancy is taken into account by implementing a hierarchy of protein families and domains. The entries at the top of these hierarchies correspond to broad families or domains that share higher level structure and/or function; the entries at the bottom correspond to specific functional subfamilies or structural /functional subclasses of domains ^30^. Using Inter-Pro for DAB clustering would require taking into account the hierarchy of protein families and domains: however, this would pose challenges of its own and would require discrimination of the functional equivalence of different signatures within the same hierarchy.

Another source of redundancy are functionally equivalent domains from distantly related sequences. Pfam represents this through related families, termed clans, where relationships may be defined by similarity of sequence, structure or profile-HMM. Clans might contain functionally equivalent domains, however it is not clear whether this is always the case as the criteria for clan definition includes functional similarity but not functional equivalence ^41^.

Members of a clan have diverging sequences and very often SB approaches would recognize the evolutionary distance between the sequences and group them in different clusters. If we were to assume that members of a clan are functionally equivalent and collect them in the same DA cluster, we will have a higher number of cases where a single DA cluster is split in multiple sequence clusters 1d→Ns. Also there would be higher number of cases of sequence clusters with the same DA but no exactly matching the DA clusters (1s→1d cases).

In many cases a one-to-one correspondence could be established between DAB and SB clusters indicating that often the sequence can be used as a proxy for function. At first this may seem a trivial result but it has a profound implication: domain model databases (in this case Pfam) contain enough information, encoded by known domain models, to represent the quasi totality of biological function encoded in the bacterial sequences analyzed here. However, it is important to stress that the comparisons have been performed considering sequences with known domains, representing currently around 85% of the genome coding content, a number that will only increase in the future.

A significant advantage of the DAB method over the SB method is that the domain architecture captured within a cluster can be used as a formal description of the function. Currently, more than 20% of all separable domains in the Pfam database, are so-called domains of unknown function (DUFs). Despite this, in bacterial species they are often essential ^42^. With the DAB method they are formally included and often semantically linked to one or more domains of known function.

The starting position of the domains was used to generate labels indicating N-C terminal order of identified domains. The labels were used only for clustering as proteins sharing the same labels were assigned to the same clusters. Choosing instead the mid-point or the C-terminal position could affect the labeling but not the obtained clusters.

A content-wise formal labeling of DAB clusters makes a seamless integration of multiple independently performed DAB analysis possible. This allows for a comparison of potential functionomes across taxonomic boundaries, as presented in Figure 7, while new genomes can be added at a computational cost *O*( *n*), with *n* the number of genomes to be analyzed. SB methods that create orthologous groups require more memory and time as they come at an *O*( *n ^2^*) computational cost. Other SB approaches, such as COGNITOR, reduce the computational costs to *O*( *n*) by using pre-computed databases. In this respect, the DAB approach is similar to the approach implemented in COGNITOR, by searching against existing databases of domains architectures. In this way the DAB approach leverages the extensive amount of work already put into defining domain families.

The bimodal shape of the distributions presented in Figure 3 and Figure 4 indicates the relative role of horizontal gene transfer and vertical descent when shaping bacterial genomes: the first peak accounts for sequences (or functions) only present in a small number of genome sequences which have been a likely acquired by horizontal gene transfer. The second peak accounts for high persistence genetic regions representing genes (or functions) belonging to the taxon core which have been likely acquired by vertical descent.

A measure of the impact of vertical descent and horizontal gene transfer is provided by the ratio between the core- and pan- genome sizes. The number of singletons provides a measure of the number of genes horizontally acquired from species outside the considered group.

Two of the most prominent differences between the two approaches are the number of retrieved singletons and the core- to pan-genome size ratio. Multiple members of the same taxon might acquire the same function through horizontal gene transfer ^43^. This is likely to occur given that they would have similar physiological characteristics, hence they would tend to occupy a similar niche or, at least, more similar than when comparing species from different taxa. As the origin of the horizontally acquired genes may vary for each organism, an SB approach will correctly recognize the heterologous origin of the corresponding sequences and those will be assigned to singletons. However, the probabilistic hidden Markov models used for domain recognition are better at recognizing the functional similarity of the considered sequences and clusters them together.

Another indication of the relative impact of horizontal and vertical gene acquisition events is provided by the openness or closedness of the genome. Values for the decay parameter *α* in Table 3 indicate a relatively large impact of horizontal gene transfer. Within the considered taxa we observed *α*_*DAB*_ > *α*_*SB*_, meaning that the sequence diversity is larger than the functional diversity: upon addition of new genomes to the sample the rate of addition of new sequence clusters appears higher than the rate of addition of new functions.

### Limitations of DAB approaches

We have shown that domain architecture-based methods can be used as an effective approach to identify clusters of functionally equivalent proteins, leading to results similar to those obtained by classical methods based on sequence similarity. However, whether DAB methods are more accurate than SB methods to assess functional equivalence will require further analysis. In this light, results of functional conservation for both approaches could be compared in terms of GO similarity and/or EC number ^44, 45^. Partial domain hits might arise as a result of alignment, annotation and sequence assembly artefacts. To reduce the number of partial domain hits additional pruning could be implemented to distinguish these cases. However, this is an open problem that requires caution as it could influence the functional capacity of an organism and clustering approaches using DA.

The performance of DAB methods may be sub-optimal when dealing with newly sequenced genomes that are not yet well-characterized enough to have all of their domains present in domain databases, since DAB methods will be unable to handle unknown architectural types. Around 15% of the genome coding content corresponds to sequences with no identified protein domains. DAB approaches can be complemented with SB methods to consider these sequences or even protein sequences with low domain coverage, possible indicating the location of protein domains yet to be identified. Since DAB methods rely on the constant upgrading of public resources like UniProt and Pfam databases, an initial assessment of domain coverage appears as a sine qua non condition for application of these methods. DAB approaches could be used to assess the consistency of existing orthologous groups in terms of their domain architectures, at least when domain architectures are expected to be completely known in advance (for instance in the case of micro-evolutionary variations within a species where mutational events may disrupt a protein’s function). For other purposes, such as the discovery of a new phyla of cellular life that contains radically different domain architectures, global similarity methods may be preferred ^45^.

## Conclusions

As protein domain databases have evolved to the point where DAB and SB approaches produce similar results in closely related organisms, the DAB approach provides a fast and efficient alternative to SB methods to identify groups of functionally equivalent/related proteins for comparative genome analysis. The lower computational cost of DAB approaches makes them the better choice for large scale comparisons involving hundreds of genomes.

Highly redundant databases, such as InterPro, are best suited for domain based protein annotation, but are not effective for DAB clustering if the goal is to identify clusters of functionally equivalent proteins. To enable DAB approaches for highly structured databases, such as Inter-Pro, the hierarchy of protein families and domains within has to be explicitly considered. Currently Pfam is for this task a better alternative.

Differences between DAB and SB approaches increase when the goal is to study bacterial groups spanning wider evolutionary distances. The functional pan-genome is more closed in comparison to the sequence based pan-genome. Both methods have a distinct approach towards horizontally transferred genes, and the DAB approach has the potential to detect functional equivalence even when sequence similarities are low.

Complementing the standardly applied sequence similarity methods with a DAB approach pinpoints potential functional protein adaptations that may add to the overall fitness.

## Data availability

The data referenced by this article are under copyright with the following copyright statement: Copyright: © 2017 Koehorst JJ et al.

Data associated with the article are available under the terms of the Creative Commons Zero "No rights reserved" data waiver (CC0 1.0 Public domain dedication). 

List of genomes used for the analysis at different phylogenetic levels. The genomes are grouped per taxonomic lineage used in this study.

**Bacillus**

*GCA*_000523045 Bacillus subtilis BEST7003

*GCA*_000782835 Bacillus subtilis

*GCA*_000832885 Bacillus thuringiensis str. Al Hakam

*GCA*_000473245 Bacillus infantis NRRL B-14911

*GCA*_000832585 Bacillus anthracis

*GCA*_000590455 Bacillus pumilus

*GCA*_000831065 Bacillus bombysepticus

*GCA*_000833275 Bacillus anthracis str. Turkey32

*GCA*_000952895 Bacillus sp.

*GCA*_000259365 Bacillus sp. JS

*GCA*_000143605 Bacillus cereus biovar anthracis str. CI

*GCA*_000186745 Bacillus subtilis BSn5

*GCA*_000987825 Bacillus methylotrophicus

*GCA*_000706725 Bacillus lehensis G1

*GCA*_000815145 Bacillus sp. Pc3

*GCA*_000496285 Bacillus toyonensis BCT-7112

*GCA*_000742855 Bacillus mycoides

*GCA*_000169195 Bacillus coagulans 36D1

*GCA*_000835145 Bacillus amyloliquefaciens KHG19

*GCA*_000321395 Bacillus subtilis subsp. subtilis str. BSP1

*GCA*_000009045 Bacillus subtilis subsp. subtilis str. 168

*GCA*_000293765 Bacillus subtilis QB928

*GCA*_000025805 Bacillus megaterium DSM 319

*GCA*_000747345 Bacillus sp. X1(2014)

*GCA*_000833005 Bacillus amyloliquefaciens

*GCA*_000408885 Bacillus paralicheniformis ATCC 9945a

*GCA*_000742895 Bacillus anthracis str. Vollum

*GCA*_000829195 Bacillus sp. OxB-1

*GCA*_000800825 Bacillus sp. WP8

*GCA*_000706705 Bacillus subtilis subsp. subtilis str. OH 131.1

*GCA*_000338735 Bacillus subtilis XF-1

*GCA*_000832445 Bacillus anthracis

*GCA*_000747335 Bacillus anthracis

*GCA*_000008505 Bacillus thuringiensis serovar konkukian str. 97-27

*GCA*_000195515 Bacillus amyloliquefaciens TA208

*GCA*_000209795 Bacillus subtilis subsp. natto BEST195

*GCA*_000017425 Bacillus cytotoxicus NVH 391-98

*GCA*_000877815 Bacillus sp. YP1

*GCA*_000177235 Bacillus cellulosilyticus DSM 2522

*GCA*_000344745 Bacillus subtilis subsp. subtilis 6051-HGW

*GCA*_000227485 Bacillus subtilis subsp. subtilis str. RO-NN-1

*GCA*_000494835 Bacillus amyloliquefaciens CC178

*GCA*_000011145 Bacillus halodurans C-125

*GCA*_000724485 Bacillus methanolicus MGA3

*GCA*_000018825 Bacillus weihenstephanensis KBAB4

*GCA*_000005825 Bacillus pseudofirmus OF4

*GCA*_000017885 Bacillus pumilus SAFR-032

*GCA*_000583065 Bacillus methylotrophicus Trigo-Cor1448

*GCA*_000349795 Bacillus subtilis subsp. subtilis str. BAB-1

*GCA*_000306745 Bacillus thuringiensis Bt407

*GCA*_000011645 Bacillus licheniformis DSM 13 = ATCC 14580

*GCA*_000497485 Bacillus subtilis PY79

*GCA*_000009825 Bacillus clausii KSM-K16

*GCA*_000227465 Bacillus subtilis subsp. spizizenii TU-B-10

*GCA*_000971925 Bacillus subtilis KCTC 1028

*GCA*_000972245 Bacillus endophyticus

*GCA*_000242895 Bacillus sp. 1NLA3E

*GCA*_000832485 Bacillus thuringiensis

*GCA*_000830075 Bacillus atrophaeus

*GCA*_000146565 Bacillus subtilis subsp. spizizenii str. W23

**Corynebacteriales**

*GCA*_000016005 Mycobacterium sp. JLS

*GCA*_000758405 Mycobacterium abscessus subsp. bolletii

*GCA*_000283295 Mycobacterium smegmatis str. MC2 155

*GCA*_001021045 Corynebacterium testudinoris

*GCA*_000341345 Corynebacterium halotolerans YIM 70093 = DSM 44683

*GCA*_000525655 Corynebacterium falsenii DSM 44353

*GCA*_000255195 Corynebacterium diphtheriae HC04

*GCA*_000523235 Nocardia nova SH22a

*GCA*_000026685 Mycobacterium leprae Br4923

*GCA*_000980815 Corynebacterium camporealensis

*GCA*_000328565 Mycobacterium sp. JS623

*GCA*_000015405 Mycobacterium sp. KMS

*GCA*_000987865 [Brevibacterium] flavum

*GCA*_001020985 Corynebacterium mustelae

*GCA*_001021065 Corynebacterium uterequi

*GCA*_000177535 Corynebacterium resistens DSM 45100

*GCA*_000011305 Corynebacterium efficiens YS-314

*GCA*_000835265 Mycobacterium avium subsp. paratuberculosis

*GCA*_000739455 Corynebacterium imitans

*GCA*_000831265 Mycobacterium kansasii 662

*GCA*_000819445 Corynebacterium humireducens NBRC 106098 = DSM 45392

*GCA*_000770235 Mycobacterium avium subsp. avium

*GCA*_000980835 Corynebacterium kutscheri

*GCA*_000010225 Corynebacterium glutamicum R

*GCA*_000590555 Corynebacterium argentoratense DSM 44202

*GCA*_000247715 Gordonia polyisoprenivorans VH2

*GCA*_000416365 Mycobacterium sp. VKM Ac-1817D

*GCA*_000418365 Corynebacterium terpenotabidum Y-11

*GCA*_000092225 Tsukamurella paurometabola DSM 20162

*GCA*_000442645 Corynebacterium maris DSM 45190

*GCA*_000277125 Mycobacterium intracellulare ATCC 13950

*GCA*_000196695 Rhodococcus equi 103S

*GCA*_000828995 Mycobacterium tuberculosis str. Kurono

*GCA*_000006605 Corynebacterium jeikeium K411

*GCA*_000022905 Corynebacterium aurimucosum

*GCA*_001021025 Corynebacterium epidermidicanis

*GCA*_000010105 Rhodococcus erythropolis PR4

*GCA*_000092825 Segniliparus rotundus DSM 44985

*GCA*_000758245 Mycobacterium bovis

*GCA*_000184435 Mycobacterium gilvum Spyr1

*GCA*_000829075 Mycobacterium avium subsp. hominissuis TH135

*GCA*_000214175 Amycolicicoccus subflavus DQS3-9A1

*GCA*_000769635 Corynebacterium ulcerans

*GCA*_000626675 Corynebacterium glyciniphilum AJ 3170

*GCA*_001026945 Corynebacterium pseudotuberculosis

*GCA*_000026445 Mycobacterium liflandii 128FXT

*GCA*_000013925 Mycobacterium ulcerans Agy99

*GCA*_000954115 Rhodococcus sp. B7740

*GCA*_000143885 Gordonia sp. KTR9

*GCA*_000014565 Rhodococcus jostii RHA1

*GCA*_000179395 Corynebacterium variabile DSM 44702

*GCA*_000732945 Corynebacterium atypicum

*GCA*_000723425 Mycobacterium marinum E11

*GCA*_000230895 Mycobacterium rhodesiae NBB3

*GCA*_000344785 Corynebacterium callunae DSM 20147

*GCA*_000010805 Rhodococcus opacus B4

*GCA*_000982715 Rhodococcus aetherivorans

*GCA*_000298095 Mycobacterium indicus pranii MTCC 9506

*GCA*_000833575 Corynebacterium singulare

*GCA*_000023145 Corynebacterium kroppenstedtii DSM 44385

**Cyanobacteria**

*GCA*_000317085 Synechococcus sp. PCC 7502

*GCA*_000011385 Gloeobacter violaceus PCC 7421

*GCA*_000014585 Synechococcus sp. CC9311

*GCA*_000012465 Prochlorococcus marinus str. NATL2A

*GCA*_000737535 Synechococcus sp. KORDI-100

*GCA*_000013205 Synechococcus sp. JA-3-3Ab

*GCA*_000021825 Cyanothece sp. PCC 7424

*GCA*_000063505 Synechococcus sp. WH 7803

*GCA*_000022045 Cyanothece sp. PCC 7425

*GCA*_000316575 Calothrix sp. PCC 7507

*GCA*_000316685 Synechococcus sp. PCC 6312

*GCA*_000012505 Synechococcus sp. CC9902

*GCA*_000317475 Oscillatoria nigro-viridis PCC 7112

*GCA*_000063525 Synechococcus sp. RCC307

*GCA*_000317695 Anabaena cylindrica PCC 7122

*GCA*_000014265 Trichodesmium erythraeum IMS101

*GCA*_000817325 Synechococcus sp. UTEX 2973

*GCA*_000737575 Synechococcus sp. KORDI-49

*GCA*_000317125 Chroococcidiopsis thermalis PCC 7203

*GCA*_000017845 Cyanothece sp. ATCC 51142

*GCA*_000020025 Nostoc punctiforme PCC 73102

*GCA*_000018105 Acaryochloris marina MBIC11017

*GCA*_000757865 Prochlorococcus sp. MIT 0801

*GCA*_000317045 Geitlerinema sp. PCC 7407

*GCA*_000012625 Synechococcus sp. CC9605

*GCA*_000737595 Synechococcus sp. KORDI-52

*GCA*_000317635 Halothece sp. PCC 7418

*GCA*_000025125 Candidatus Atelocyanobacterium thalassa isolate ALOHA

*GCA*_000010625 Microcystis aeruginosa NIES-843

*GCA*_000317065 Pseudanabaena sp. PCC 7367

*GCA*_000312705 Anabaena sp. 90

*GCA*_000316515 Cyanobium gracile PCC 6307

*GCA*_000316605 Leptolyngbya sp. PCC 7376

*GCA*_000317025 Pleurocapsa sp. PCC 7327

*GCA*_000009705 Nostoc sp. PCC 7120

*GCA*_000013225 Synechococcus sp. JA-2-3B’a(2–13)

*GCA*_000757845 Prochlorococcus sp. MIT 0604

*GCA*_000317515 Microcoleus sp. PCC 7113

*GCA*_000734895 Calothrix sp. 336/3

*GCA*_000007925 Prochlorococcus marinus subsp. marinus str. CCMP1375

*GCA*_000021805 Cyanothece sp. PCC 8801

*GCA*_000019485 Synechococcus sp. PCC 7002

*GCA*_000317655 Cyanobacterium stanieri PCC 7202

*GCA*_000316625 Nostoc sp. PCC 7107

*GCA*_000011465 Prochlorococcus marinus subsp. pastoris str. CCMP1986

*GCA*_000316665 Rivularia sp. PCC 7116

*GCA*_000317105 Oscillatoria acuminata PCC 6304

*GCA*_000317435 Calothrix sp. PCC 6303

*GCA*_000317555 Gloeocapsa sp. PCC 7428

*GCA*_000478825 Synechocystis sp. PCC 6714

*GCA*_000204075 Anabaena variabilis ATCC 29413

*GCA*_000317575 Stanieria cyanosphaera PCC 7437

*GCA*_000161795 Synechococcus sp. WH 8109

*GCA*_000011345 Thermosynechococcus elongatus BP-1

*GCA*_000317615 Dactylococcopsis salina PCC 8305

*GCA*_000284135 Synechocystis sp. PCC 6803 substr. GT-I

*GCA*_000024045 Cyanothece sp. PCC 8802

*GCA*_000317495 Crinalium epipsammum PCC 9333

*GCA*_000317675 Cyanobacterium aponinum PCC 10605

*GCA*_000012525 Synechococcus elongatus PCC 7942

**Enterobacteriaceae**

*GCA*_000259175 Providencia stuartii MRSN 2154

*GCA*_000214805 Serratia sp. AS13

*GCA*_000330865 Serratia marcescens FGI94

*GCA*_001010285 Photorhabdus temperata subsp. thracensis

*GCA*_000364725 Candidatus Moranella endobia PCVAL

*GCA*_000521525 Buchnera aphidicola str. USDA (Myzus persicae)

*GCA*_000517405 Candidatus Sodalis pierantonius str. SOPE

*GCA*_000012005 Shigella dysenteriae Sd197

*GCA*_000196475 Photorhabdus asymbiotica

*GCA*_000750295 Salmonella enterica subsp. enterica serovar Enteritidis

*GCA*_000007885 Yersinia pestis biovar Microtus str. 91001

*GCA*_000739495 Klebsiella pneumoniae

*GCA*_000252995 Salmonella bongori NCTC 12419

*GCA*_000270125 Pantoea ananatis AJ13355

*GCA*_000215745 Enterobacter aerogenes KCTC 2190

*GCA*_000092525 Shigella sonnei Ss046

*GCA*_000020865 Edwardsiella tarda EIB202

*GCA*_000023545 Dickeya dadantii Ech703

*GCA*_000238975 Serratia symbiotica str. ’Cinara cedri’

*GCA*_000975245 Serratia liquefaciens

*GCA*_000006645 Yersinia pestis KIM10+

*GCA*_000224675 Enterobacter asburiae LF7a

*GCA*_000007405 Shigella flexneri 2a str. 2457T

*GCA*_001022275 Citrobacter freundii

*GCA*_000963575 Klebsiella michiganensis

*GCA*_000504545 Cronobacter sakazakii CMCC 45402

*GCA*_000012025 Shigella boydii Sb227

*GCA*_000814125 Enterobacter cloacae

*GCA*_000987925 Yersinia enterocolitica

*GCA*_000011745 Candidatus Blochmannia pennsylvanicus str. BPEN

*GCA*_000255535 Rahnella aquatilis HX2

*GCA*_000952955 Escherichia coli

*GCA*_000695995 Serratia sp. FS14

*GCA*_000648515 Citrobacter freundii CFNIH1

*GCA*_001022295 Klebsiella oxytoca

*GCA*_000147055 Dickeya dadantii 3937

*GCA*_000348565 Edwardsiella piscicida C07-087

*GCA*_000742755 Klebsiella pneumoniae subsp. pneumoniae

*GCA*_000027225 Xenorhabdus bovienii SS-2004

*GCA*_000247565 Wigglesworthia glossinidia endosymbiont of Glossina morsitans morsitans (Yale colony)

*GCA*_000828815 Candidatus Tachikawaea gelatinosa

*GCA*_000022805 Yersinia pestis D106004

*GCA*_001006005 Serratia fonticola

*GCA*_000018625 Salmonella enterica subsp. arizonae serovar 62:z4,z23:-

*GCA*_000478905 Candidatus Pantoea carbekii

*GCA*_000410515 Enterobacter sp. R4-368

*GCA*_000148935 Pantoea vagans C9-1

*GCA*_000444425 Proteus mirabilis BB2000

*GCA*_000747565 Serratia sp. SCBI

*GCA*_001022135 Kluyvera intermedia

*GCA*_000757825 Cedecea neteri

*GCA*_000294535 Pectobacterium carotovorum subsp. carotovorum PCC21

*GCA*_000834375 Yersinia pseudotuberculosis YPIII

*GCA*_000043285 Candidatus Blochmannia floridanus

*GCA*_000093065 Candidatus Riesia pediculicola USDA

*GCA*_000834515 Yersinia intermedia

*GCA*_000759475 Pantoea rwandensis

*GCA*_000027065 Siccibacter turicensis z3032

*GCA*_000582515 Yersinia similis

*GCA*_000300455 Kosakonia sacchari SP1

**Helicobacter pylori**

*GCA*_000148855 Helicobacter pylori SJM180

*GCA*_000021165 Helicobacter pylori G27

*GCA*_000185245 Helicobacter pylori SouthAfrica7

*GCA*_000093185 Helicobacter pylori v225d

*GCA*_000277365 Helicobacter pylori Shi417

*GCA*_000498315 Helicobacter pylori BM012A

*GCA*_000270065 Helicobacter pylori F57

*GCA*_000392455 Helicobacter pylori UM032

*GCA*_000277385 Helicobacter pylori Shi169

*GCA*_000008525 Helicobacter pylori 26695

*GCA*_000270045 Helicobacter pylori F32

*GCA*_000148915 Helicobacter pylori Sat464

*GCA*_000185225 Helicobacter pylori Lithuania75

*GCA*_000600045 Helicobacter pylori oki102

*GCA*_000600205 Helicobacter pylori oki828

*GCA*_000192335 Helicobacter pylori 2018

*GCA*_000827025 Helicobacter pylori

*GCA*_000590775 Helicobacter pylori SouthAfrica20

*GCA*_000270025 Helicobacter pylori F30

*GCA*_000148665 Helicobacter pylori 908

*GCA*_000392515 Helicobacter pylori UM037

*GCA*_000392475 Helicobacter pylori UM299

*GCA*_000262655 Helicobacter pylori XZ274

*GCA*_000008785 Helicobacter pylori J99

*GCA*_000685745 Helicobacter pylori

*GCA*_000185205 Helicobacter pylori Gambia94/24

*GCA*_000826985 Helicobacter pylori 26695-1

*GCA*_000315955 Helicobacter pylori Aklavik117

*GCA*_000498335 Helicobacter pylori BM012S

*GCA*_000277405 Helicobacter pylori Shi112

*GCA*_000224535 Helicobacter pylori Puno120

*GCA*_000317875 Helicobacter pylori Aklavik86

*GCA*_000600185 Helicobacter pylori oki673

*GCA*_000196755 Helicobacter pylori B8

*GCA*_000439295 Helicobacter pylori UM298

*GCA*_000348885 Helicobacter pylori OK310

*GCA*_000307795 Helicobacter pylori 26695

*GCA*_000013245 Helicobacter pylori HPAG1

*GCA*_000392535 Helicobacter pylori UM066

*GCA*_000185185 Helicobacter pylori India7

*GCA*_000213135 Helicobacter pylori 83

*GCA*_000685705 Helicobacter pylori

*GCA*_000224575 Helicobacter pylori SNT49

*GCA*_000600085 Helicobacter pylori oki112

*GCA*_000023805 Helicobacter pylori 52

*GCA*_000348865 Helicobacter pylori OK113

*GCA*_000259235 Helicobacter pylori HUP-B14

*GCA*_000020245 Helicobacter pylori Shi470

*GCA*_000270005 Helicobacter pylori F16

*GCA*_000192315 Helicobacter pylori 2017

*GCA*_000685665 Helicobacter pylori

*GCA*_000600165 Helicobacter pylori oki422

*GCA*_000255955 Helicobacter pylori ELS37

*GCA*_000021465 Helicobacter pylori P12

*GCA*_000600145 Helicobacter pylori oki154

*GCA*_000224555 Helicobacter pylori Puno135

*GCA*_000011725 Helicobacter pylori 51

*GCA*_000148895 Helicobacter pylori Cuz20

*GCA*_000817025 Helicobacter pylori

*GCA*_000178935 Helicobacter pylori 35A

**Listeria monocytogenes**

*GCA*_000438745 Listeria monocytogenes

*GCA*_000438705 Listeria monocytogenes

*GCA*_001027125 Listeria monocytogenes

*GCA*_000438725 Listeria monocytogenes

*GCA*_000197755 Listeria monocytogenes

*GCA*_001027245 Listeria monocytogenes

*GCA*_001027085 Listeria monocytogenes

*GCA*_001005925 Listeria monocytogenes

*GCA*_000746625 Listeria monocytogenes

*GCA*_000382925 Listeria monocytogenes

*GCA*_000438665 Listeria monocytogenes

*GCA*_000800335 Listeria monocytogenes

*GCA*_001027165 Listeria monocytogenes

*GCA*_000438605 Listeria monocytogenes

*GCA*_000438585 Listeria monocytogenes

*GCA*_000808055 Listeria monocytogenes

*GCA*_000950775 Listeria monocytogenes

*GCA*_001027065 Listeria monocytogenes

*GCA*_000600015 Listeria monocytogenes

*GCA*_001027205 Listeria monocytogenes

*GCA*_000438685 Listeria monocytogenes

*GCA*_001005985 Listeria monocytogenes

*GCA*_000438625 Listeria monocytogenes

*GCA*_000681515 Listeria monocytogenes

*GCA*_000438645 Listeria monocytogenes

*GCA*_000210815 Listeria monocytogenes

**Pseudomonas**

*GCA*_000829885 Pseudomonas aeruginosa

*GCA*_000510285 Pseudomonas monteilii SB3078

*GCA*_000988485 Pseudomonas syringae pv. syringae B301D

*GCA*_000013785 Pseudomonas stutzeri A1501

*GCA*_000759535 Pseudomonas cremoricolorata

*GCA*_000953455 Pseudomonas pseudoalcaligenes

*GCA*_000981825 Pseudomonas aeruginosa

*GCA*_000661915 Pseudomonas stutzeri

*GCA*_000508205 Pseudomonas sp. TKP

*GCA*_000014625 Pseudomonas aeruginosa UCBPP-PA14

*GCA*_000019445 Pseudomonas putida W619

*GCA*_000316175 Pseudomonas sp. UW4

*GCA*_000498975 Pseudomonas mosselii SJ10

*GCA*_000473745 Pseudomonas aeruginosa VRFPA04

*GCA*_000691565 Pseudomonas putida

*GCA*_000730425 Pseudomonas fluorescens

*GCA*_000007805 Pseudomonas syringae pv. tomato str. DC3000

*GCA*_000349845 Pseudomonas denitrificans ATCC 13867

*GCA*_000026105 Pseudomonas entomophila L48

*GCA*_000689415 Pseudomonas knackmussii B13

*GCA*_000325725 Pseudomonas putida HB3267

*GCA*_000412695 Pseudomonas resinovorans NBRC 106553

*GCA*_000831585 Pseudomonas plecoglossicida

*GCA*_000756775 Pseudomonas sp. 20_BN

*GCA*_000590475 Pseudomonas stutzeri

*GCA*_000829255 Pseudomonas aeruginosa

*GCA*_000761155 Pseudomonas rhizosphaerae

*GCA*_001038645 Pseudomonas stutzeri

*GCA*_000264665 Pseudomonas putida ND6

*GCA*_000007565 Pseudomonas putida KT2440

*GCA*_000494915 Pseudomonas sp. VLB120

*GCA*_000226155 Pseudomonas aeruginosa M18

*GCA*_000213805 Pseudomonas fulva 12-X

*GCA*_000194805 Pseudomonas brassicacearum subsp. brassicacearum NFM421

*GCA*_000336465 Pseudomonas poae RE*1-1-14

*GCA*_000828695 Pseudomonas protegens Cab57

*GCA*_000800255 Pseudomonas parafulva

*GCA*_000257545 Pseudomonas mandelii JR-1

*GCA*_000012205 Pseudomonas savastanoi pv. phaseolicola 1448A

*GCA*_000816985 Pseudomonas aeruginosa

*GCA*_000746525 Pseudomonas alkylphenolia

*GCA*_000496605 Pseudomonas aeruginosa PA1

*GCA*_000204295 Pseudomonas mendocina NK-01

*GCA*_000829415 Pseudomonas sp. StFLB209

*GCA*_000012265 Pseudomonas protegens Pf-5

*GCA*_000412675 Pseudomonas putida NBRC 14164

*GCA*_000397205 Pseudomonas protegens CHA0

*GCA*_000648735 Pseudomonas syringae pv. actinidiae ICMP 18884

*GCA*_000012245 Pseudomonas syringae pv. syringae B728a

*GCA*_000761195 Pseudomonas chlororaphis subsp. aurantiaca

*GCA*_000818015 Pseudomonas balearica DSM 6083

*GCA*_000219605 Pseudomonas stutzeri ATCC 17588 = LMG 11199

*GCA*_000219705 Pseudomonas putida S16

*GCA*_000511325 Pseudomonas sp. FGI182

*GCA*_000508765 Pseudomonas aeruginosa LES431

*GCA*_000297075 Pseudomonas pseudoalcaligenes CECT 5344

*GCA*_000517305 Pseudomonas cichorii JBC1

*GCA*_000963835 Pseudomonas chlororaphis

*GCA*_000327065 Pseudomonas stutzeri RCH2

*GCA*_000271365 Pseudomonas aeruginosa DK2

**Streptococcus**

*GCA*_000211015 Streptococcus pneumoniae SPN034183

*GCA*_000210975 Streptococcus pneumoniae INV104

*GCA*_000203195 Streptococcus gallolyticus subsp. gallolyticus ATCC BAA-2069

*GCA*_001020185 Streptococcus pyogenes

*GCA*_000253155 Streptococcus oralis Uo5

*GCA*_000696505 Streptococcus equi subsp. zooepidemicus CY

*GCA*_000463355 Streptococcus intermedius B196

*GCA*_000698885 Streptococcus thermophilus ASCC 1275

*GCA*_000014205 Streptococcus sanguinis SK36

*GCA*_000007045 Streptococcus pneumoniae R6

*GCA*_000306805 Streptococcus intermedius JTH08

*GCA*_000196595 Streptococcus pneumoniae TCH8431/19A

*GCA*_000262145 Streptococcus parasanguinis FW213

*GCA*_001026925 Streptococcus agalactiae

*GCA*_000251085 Streptococcus pneumoniae ST556

*GCA*_000019025 Streptococcus pneumoniae Taiwan19F-14

*GCA*_000211055 Streptococcus pneumoniae SPN994039

*GCA*_000688775 Streptococcus sp. VT 162

*GCA*_000231905 Streptococcus suis D12

*GCA*_000026665 Streptococcus pneumoniae ATCC 700669

*GCA*_000283635 Streptococcus macedonicus ACA-DC 198

*GCA*_000014365 Streptococcus pneumoniae D39

*GCA*_000019265 Streptococcus pneumoniae Hungary19A-6

*GCA*_000299015 Streptococcus pneumoniae gamPNI0373

*GCA*_000019985 Streptococcus pneumoniae CGSP14

*GCA*_000463395 Streptococcus constellatus subsp. pharyngis C232

*GCA*_000187935 Streptococcus parauberis NCFD 2020

*GCA*_000253315 Streptococcus salivarius JIM8777

*GCA*_000427055 Streptococcus agalactiae ILRI112

*GCA*_000246835 Streptococcus infantarius subsp. infantarius CJ18

*GCA*_000427075 Streptococcus agalactiae ILRI005

*GCA*_000007465 Streptococcus mutans UA159

*GCA*_000831165 Streptococcus anginosus

*GCA*_000147095 Streptococcus pneumoniae 670-6B

*GCA*_000817005 Streptococcus pneumoniae

*GCA*_000180515 Streptococcus pneumoniae SPNA45

*GCA*_000441535 Streptococcus lutetiensis 033

*GCA*_000210955 Streptococcus pneumoniae OXC141

*GCA*_000009545 Streptococcus uberis 0140J

*GCA*_000648555 Streptococcus iniae

*GCA*_000027165 Streptococcus mitis B6

*GCA*_000018985 Streptococcus pneumoniae JJA

*GCA*_000270165 Streptococcus pasteurianus ATCC 43144

*GCA*_000479315 Streptococcus sp. I-P16

*GCA*_000478925 Streptococcus anginosus subsp. whileyi MAS624

*GCA*_000019825 Streptococcus pneumoniae G54

*GCA*_000017005 Streptococcus gordonii str. Challis substr. CH1

*GCA*_000479335 Streptococcus sp. I-G2

*GCA*_000385925 Streptococcus oligofermentans AS 1.3089

*GCA*_000210935 Streptococcus pneumoniae INV200

*GCA*_000211035 Streptococcus pneumoniae SPN994038

*GCA*_000221985 Streptococcus pseudopneumoniae IS7493

*GCA*_000006885 Streptococcus pneumoniae TIGR4

*GCA*_000018965 Streptococcus pneumoniae 70585

*GCA*_000348705 Streptococcus pneumoniae PCS8235

*GCA*_000210995 Streptococcus pneumoniae SPN034156

*GCA*_000231925 Streptococcus suis ST1

*GCA*_000019005 Streptococcus pneumoniae P1031

*GCA*_000188715 Streptococcus dysgalactiae subsp. equisimilis ATCC 12394

*GCA*_000026585 Streptococcus equi subsp. equi 4047

## Figures and Tables

**Figure S1.**
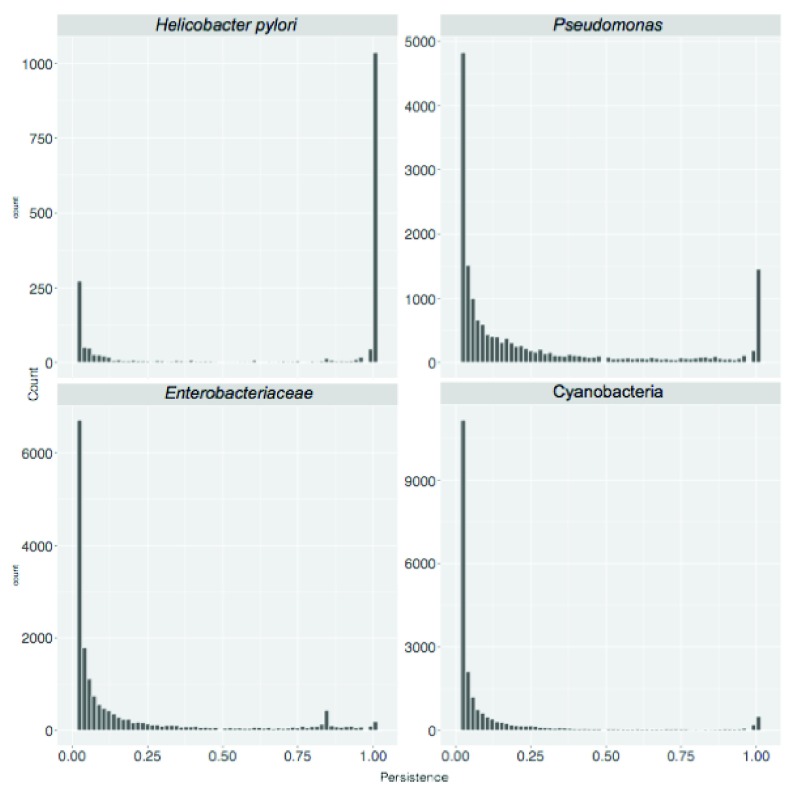
Persistence of Sequence Based (SB) clusters. Cluster persistence is defined as the relative number of genomes with at least one protein assigned to the cluster. The plots show frequency of SB clusters according to their persistence. Publicly available and complete genome sequences assigned to each taxon were selected so that phylogenetic diversity within the taxon was preserved, as described in materials and methods. 60 distinct genome sequences were considered for each taxon shown.

**Figure S2.**
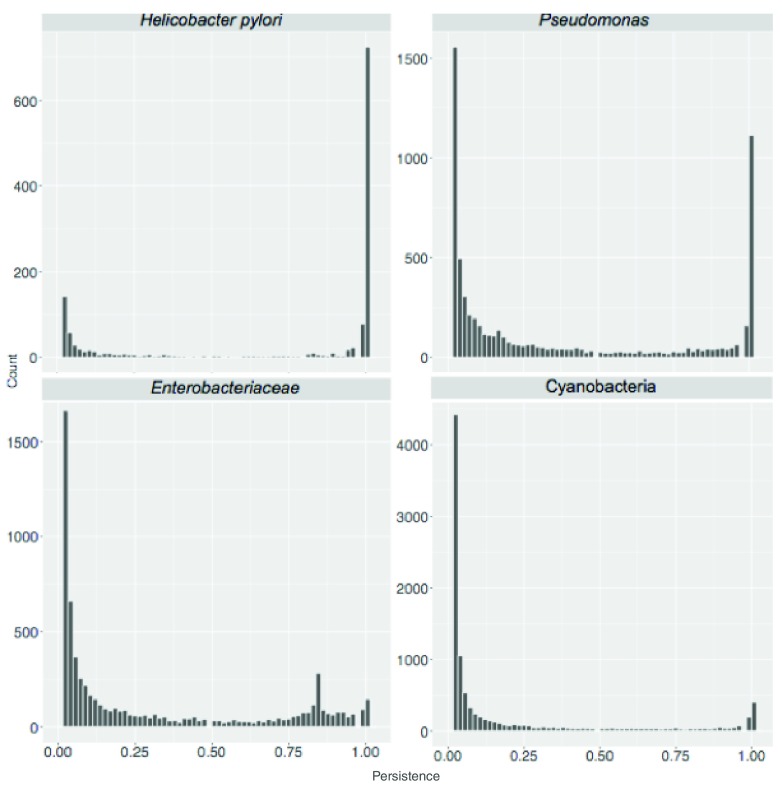
Persistence of Domain Architecture Based (DAB) clusters. The plots show frequency of DAB clusters according to their persistence.

**Figure S3.**
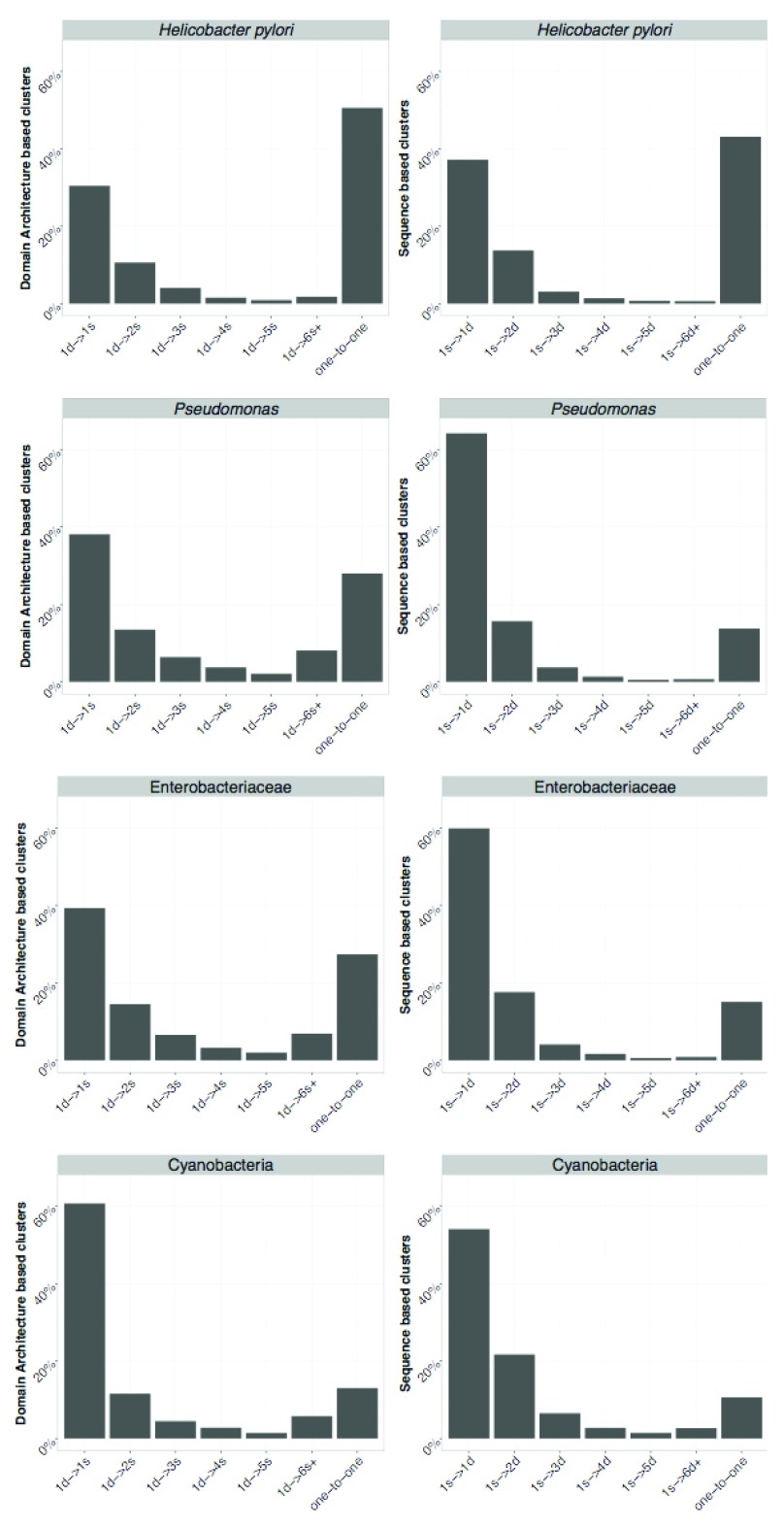
Comparison between DAB and SB clusters. On the left DAB is used as a reference and each bar represents the relative frequency of one DAB cluster containing sequences assigned to {1, 2, . . . , 5} and 6 or more SB clusters and one-to-one represents the relative frequency of identical cluster. Similarly, on the right SB is used as a reference. Axis labels follow notation in Figure 5
