# Supplementary material for: Protein domain architectures provide a fast, efficient and scalable alternative to sequence-based methods for comparative functional genomics
Source: F1000Res. 2017 Jun 27;5:1987. Originally published 2016 Aug 15. [Version 3] doi: 10.12688/f1000research.9416.3 (PMC5031134; doi:10.12688/f1000research.9416.3)
Supplement: Peer review history

# Peer review: Referee response for version 2

**DOI:** 10.5256/f1000research.10932.r17969

**Published:** 2017-05-08

**Authors:** David M. Kristensen

**Affiliations:** 1 Department of Biomedical Engineering, The University of Iowa, Iowa City, IA, USA

The manuscript is overall of much higher quality than it was previously. However, not all of the comments were addressed fully. The most egregious of these oversights is that it still gives the impression that a “straw man” argument is being set up to easily fall when set up against the authors’ favored approach. Fortunately this is not quite the case, and therefore this is likely an entirely unintentional effect of not clearly explaining the method that is being used or its comparison to other methods, but even so this issue is quite important since it can easily mislead an unwary reader. e.g., statements about the time and memory requirements of the SB approach having to scale quadratically with the number of genomes to be compared are untrue. In fact, while SB methods to construct orthologous groups do often take advantage of a full all-against-all comparison (and therefore these methods require a quadratic scale), perhaps the more proper comparison of DAB is not to the set of SB methods that construct orthologous groups, but rather to those that extend existing groups into new genomes (much like DAB does not construct domain families, but merely extends these existing families into new genomes, taking into account their architectures while this is being done). This fact is now acknowledged in the manuscript, but is buried deeply in the middle of the Discussion section, and yet the confusing description of the comparison of the quadratic to linear scales also remains at several places in the manuscript (such as in the abstract and the second paragraph of the introduction). Perhaps this was merely an oversight, but in any case this issue should be made much more clear than it currently is. If I understand things correctly, the overall summary seems to be that: the DAB approach, much like several existing SB approaches, leverages the extensive amount of work (much of it done with manual curation) already put into defining domain families, and attempts to extend these families to identify new members of orthologous groups in newly discovered genomes - which it is able to do more accurately than similar SB methods due to taking into account domain architectures. Both of these approaches - DAB and SB - scale linearly with the number of new genomes to be compared. In contrast, there is also a different class of SB methods (such as OrthaGogue, and COGs) that create orthologous groups de novo - these methods require more memory and time since they scale quadratically with the number of new genomes, although this class of method provides the advantage of being able to work even in the absence of domain family information, which DAB is not able to do.

 Another oversight occurs in the sentence that “Our aim was to investigate whether using HMMs instead of sequence similarity would yield similar results”, where instead of HMM I think the authors meant domain architectures? (and since domain architecture comparisons also rely on sequence similarity, perhaps also add the word “alone” after “sequence similarity” to distinguish the use of the latter alone vs. in combination with domain architectures)

I have read this submission. I believe that I have an appropriate level of expertise to confirm that it is of an acceptable scientific standard, however I have significant reservations, as outlined above.

# Response

**Published:** 2017-06-23

**Authors:** Jasper Koehorst

Thanks, we have amended the manuscript as suggested by the reviewer. 

Specifically:

We have deleted from the abstract and introduction the statements about the time and memory requirements of the SB approach having to scale quadratically with the number of genomes. 

We have modified the paragraph in the discussion to further emphasizes that  DAB is similar to SB methods that extend existing groups into new genomes. We have also rephase the reviewers comment on the extensive use DAB does on the amount of work put on defining domain families as we think it might  further clarify the text. 

The sentence “Our aim was to investigate whether using HMMs instead of sequence similarity would yield similar results” has been modified as suggested to: “Our aim was to investigate whether using domain architectures instead of sequence similarity alone would yield similar results.”

# Peer review: Referee response for version 1

**DOI:** 10.5256/f1000research.10140.r15678

**Published:** 2016-09-15

**Authors:** David M. Kristensen

**Affiliations:** 1 Department of Biomedical Engineering, The University of Iowa, Iowa City, IA, USA

The limitations of global sequence similarity based methods to identify proteins that perform similar functions are well-known. Thus, the approach described in this manuscript of using domain-based clustering of orthologous groups (DAB) represents an exciting and very welcome addition to the field. Or at least it will when it is fully developed, although this manuscript has not convinced me that it outperforms other methods at its current level of development, and I have several substantial reservations about some of its content:

- As the first reviewer also mentioned, methods such as CDART and DELTA-BLAST (published in 2002 and 2012, respectively) have been available for many years. The latter even seems to aim to perform the exact same function as DAB, by considering domain architectures. How is DAB different or better? I suspect that DAB may have greater accuracy since it uses HMMs rather than PSSMs, but this remains to be shown, and DELTA-BLAST is far easier for a user to run, since it is available as a webserver.
- The comparison performed in this manuscript appears to fall prey to a straw man argument. In some cases, but not all, re-writing the relevant sections of the manuscript would help to avoid any misconceptions in this regard.

a) The issue of replacing a *O(n ^2^)* cost with a *O(n)* one upon addition of a new genome was dealt with over 15 years ago, so the statement "On the other hand, addition of a new genome using an SB approach require a new set of all-against-all sequence comparisons which come at a *O(n ^2^)* computational cost" is false - at least as it is currently written. It is true that building groups of orthologs do require an initial *O(n ^2^)* computational cost, but once those orthologous groups are formed, methods such as COGNITOR (first published in the year 2000) work extremely quickly and efficiently to assign genes in newly-sequenced genomes to existing groups. In fact, COGNITOR works in the exact same manner in which DAB uses pre-computed domain databases to achieve the much lower *O(n)* cost, although in COGNITOR's case it searches against a pre-computed database of orthologous groups (of which there are far fewer than domains, so with a smaller "n" it would actually be faster than DAB).

 In should be noted that despite DAB's somewhat higher cost, it has the theoretical potential to achieve better accuracy than COGNITOR (at least in some cases) since as a global sequence similarity approach, the latter does not explicitly consider domain architecture. At least not in an automated fashion - doing so would require manual curation of its results, which is often done by careful researchers, but is not a process that is scalable to handle the ever-decreasing cost and ever-increasing amounts of genomic data. Although since a comparison with COGNITOR was not included in the manuscript, either in terms of speed or accuracy, it is unknown how much more useful DAB would be in practice.

 b) Even the initial *O(n ^2^)* cost does not have to be terribly burdensome, since the SIMAP method pre-computes and stores BLAST results between all pairs of sequenced genomes anyway, and then uses efficient database retrieval methods to report the stored results. When a new genome is added, *O(n)* new comparisons have to be made - for a total accumulated cost of *O(n ^2^)*, although with the work spread out over many years - and these in turn are useful for many other purposes, thus mitigating the construction costs. For instance, the EGGNOG database uses this method to build groups of orthologs.

 c) Why was only a single SB method chosen to be a representative for this entire class of approaches? Multiple forms of DAP were tested, whereas the only SB method used for comparison was one that uses a strict e-value cutoff of 1e-5, in the form of OrthaGogue and the OrthoMCL method. Also, why was the latter chosen to be this single representative? The latter approach was designed (nearly a decade and a half ago) for eukaryotic organisms, and while it has been applied more recently to bacteria as well, it is by no means the only - or even necessarily the best - approach for prokaryotic genomes. One advantage that it has is that it is completely automated, and thus is "easy" for people to use (even if, as this manuscript points out, horribly slow due to the *O(n ^2^)* procedure that it uses). On the other hand, methods like CDART and COGTRIANGLES are all also automated (the latter of which uses no arbitrary e-value cutoff - that is, the results are robust to e-values over an immense range such as 1e-5, 1, 10, or even well beyond that on up to 100, or even 1000), and some pre-computed databases (such as COGs, representing the protein families present in the last common ancestor of all cellular life several billions of years ago) even take advantage of further manual validation, and from which pre-computed groups can be identified in newly-sequenced genomes by the fully automated and even easier approaches such as DELTA-BLAST and COGNITOR. Is it at least possible that the poorer performance of SB methods in comparison to DAB as shown in the current manuscript is due to the choice of this particular SB method? I for one would have loved to see a comparison against the new release of the COGs database last year, since due to its being manually curated it acts as a sort of "Gold Standard" that can be tested against, with perhaps the EGGNOG groups being used as a more realistic measure of what a purely automated method can do without human supervision. Likely, DAB would fall somewhere in-between, and which would benefit the community of researchers who want to do comparative genomics of prokaryotic organisms to have a fully automated method that was demonstrated to surpass the existing fully automated methods. As it now stands though, DAB has only been shown to surpass OrthoMCL, which is not hard to do at all. Indeed, as seventh paragraph of the Discussion section (starting "Two of the most prominent...") states, unlike DAB, the SB methods were not able to cluster together the proteins with functional similarity but little sequence identity, especially across wider taxonomic ranges - which of course is what would be expected from a SB method that uses an e-value cutoff of 1e-5.

 d) Above and beyond the choice of SB method, it also seems that there may have been a bug in its implementation. The statement "For SB clustering we also observed the case of identical protein sequences not clustered together, probably because of the tie breaking implementation when BBH are scored." However, this was not supposed to happen, due to the within-species reciprocal BBH procedure that is used. In contrast, the tie breaking refers to between-species comparisons, but as shown in Figure 1 of the OrthoMCL paper (http://www.ncbi.nlm.nih.gov/pubmed/12952885), these two sources of information were supposed to have been combined together to form the final orthologous groups. If the proteins were highly similar (e.g., 99%) then perhaps a tie-breaking could be explained, but for 100% identical proteins - e.g., produced by a tanden duplication event - then they should have been collected into the group. One possibility is that this particular SB method simply was not designed to handle the large numbers of extremely closely-related genome assemblies that are available today, since at the time, very few instances of multiple genomic assemblies were available for the same species. If this explanation was demonstrated to be the reason why these identical proteins were not clustered together, that would be another reason for a user to choose to use DAB over this particular SB method. In any case (bug, design flaw, or something else), this event could greatly contribute to explaining some of the results that were observed whereby this single SB method found so many more singletons than DAB with Pfam - i.e., fixing the bug, or using some other SB method, may move many of those singletons into clusters. Although it would not explain why DAB with InterPro found even more singletons than this SB method?

  

- DAB has a lot of potential, but its limitations need to be made more clear:

a) Why and how is the matrix of domain architecture binarized? Specifically, what if multiple copies of a domain are present? And does order matter - such as the architectures shown in Figure 2 of "A+B" and "B+A"? So, would "B+A+A" be a different architecture? And, as another reviewer also pointed out, what about "complicated" domain topologies where domains are interrupted by the insertion of another domain? Another major aspect of partial topologies is if DAB only recognizes some but not all of a newly-discovered architecture. E.g., a protein with architecture A+B+C+D, where A is known but B, C and D domains are not yet known. How would this be handled by DAB? Would it be reduced to appear merely as a single-domain "A" architecture? If so, how could that be distinguished from an architecture such as A+Z, which would also be reduced to appear just as a single-domain A? It seems like global sequence similarity methods might be more useful in those particular scenarios? i.e., if all the above domains were the same length, and a coverage threshold was used, then A+B+C+D could not be put into the same group as A+Z and A. Therefore, DAB seems primarily useful to either quickly extend known architectural types into a newly sequenced genome, but at the cost of not being able to work with unknown types.

 b) For newly sequenced genomes that are not yet well-characterized enough to have all of their domains present in the domain databases, DAB can be severely handicapped in comparison to global sequence similarity methods that do not have this limitation. In particular, Table 1 shows that up to nearly a fifth of the *H. pylori* and Cornebacteriales genomes are not able to be assigned to domain families. Even these numbers are merely lower-bound estimates, since brand-new architectures are expected to be discovered constantly, and yet these may incorporate at least one element that is known - such as the aforementioned A+B+C+D architecture, where only the A domain is represented in Pfam, but B and C and D are unknown. And yet it seems likely that even the fact that these domains are unknown would go unrecognized by the DAB approach - unless a factor is added to look for large segments of a gene that do not have matches in the databases of known domains. Therefore, the cost of DAB not being able to work with unknown architectural types might be quite high indeed. Worse, the exact value of that cost is also likewise unknown, and yet it would seem to be the single crucial piece of information that is most sorely needed in order to answer the question: does the benefits of DAB outweigh its costs?

  

- If the goal is to bring together groups of proteins that have functional equivalence, then why was the only comparison that was done performed against the presence/absence membership of SB orthology approaches? Would it not have been better to actually measure the functional consistency observed within the SB groups, and within the DAB groups, in order to show that the latter was higher than the former? Many other methods that purport to improve upon the state-of-the-art orthology prediction process do just that - for instance Figure 4 of http://www.ncbi.nlm.nih.gov/pubmed/19148271 shows several comparisons with similarity of GO terms, enzyme nomenclature (EC), gene expression, and syntenic local neighborhood tests, with 12 different methods of orthology prediction. While neighborhood conservation is irrelevant for the issue of functional equivalence, the former three (or at least GO terms) would help to answer whether DAB is truly better than SB at the task of measuring functional equivalence. It would also help to answer whether this improved functional equivalence would be outweighed by the costs of being unable to handle unknown domain architectures, especially for highly divergent new genomes. If not, DAB may still be useful to check the consistency of existing orthologous groups in terms of their architecture, at least when domain architectures are expected to be completely known in advance - e.g., microevolutionary variations within a species where mutational events may disrupt a protein's function - but for other tasks such as the discovery of a new phyla of cellular life that contains radically different domain architectures, global similarity methods may be preferable instead.

    

 Finally, some minor points concerning Figure 2:

1. the vertical arrows seem to be pointing the wrong direction - a gene sequence undoubtebly contains more information content than a mere functional description. e.g., if I were to give you a GO code for molecular function, or biological process, then I could not tell you whether the original gene sequence is closer to one type of bacteria vs another type; but if I had the original gene sequence, then I could answer that question as well as many more.
2. I did not see a description of how amino acid coordinates are used anywhere else in the manuscript, either in DAB itself or in the comparison? In short, what does "Structure" have to do with anything, other than the general theoretical flow of "sequence begets structure which begets function"? If the purpose of Figure 2 is to describe the flowchart of DAB specifically though, it should focus only on the relevant elements. I suppose Structure could have meant how the sequence alignment was made, but if that were true, then DAB would only work for domain families for which a structure is available, instead of those for which only genomic or individual gene sequence has been provided.
3. The ordering also seems unclear - wouldn't BBHs inform HMM domains, which then in turn inform domain architectures? Or if starting with BBHs, then how could architectures possibly be known prior to knowing the domains themselves? Or if it should be read from top to bottom as shown, how exactly does one start with Function (e.g., a GO term) and then, somehow via Structure, thereby arrive at a Sequence alignment? Specifically, is a Pfam entry a "Function", from which the Sequence alignment is downloaded? Or are Function and the Sequence alignment both part of the starting Pfam entry (and then again, what does any of that have to do with Structure)? From which domains are found (but aren't Pfam entries domains to begin with?), and then BBHs are made from the domain architectures? (an extremely different way of doing the BBH procedure, which is normally done via Sequence alignments). In any case, as pointed out by other reviewers, the methodology used by DAB is not clearly explained in this figure, nor in the manuscript text.

Also, the last paragraph of the Discussion uses the word "closeness", but I think "closedness" was intended.

I have read this submission. I believe that I have an appropriate level of expertise to confirm that it is of an acceptable scientific standard, however I have significant reservations, as outlined above.

# Response

**Published:** 2016-11-09

**Authors:** Jasper Koehorst

1. As the first reviewer also mentioned, methods such as CDART and DELTA-BLAST (published in 2002 and 2012, respectively) have been available for many years. The latter even seems to aim to perform the exact same function as DAB, by considering domain architectures. How is DAB different or better? I suspect that DAB may have greater accuracy since it uses HMMs rather than PSSMs, but this remains to be shown, and DELTA-BLAST is far easier for a user to run, since it is available as a webserver.
**Following the suggestions made by the other reviewers we have added a paragraph in the Introduction regarding domain architectures, comparison of domain architectures and their use for sequence search. We have also discussed on how these have been included in domain databases and on the preservation of domain architectures at high phylogenetic distances.    **
**We agree that most likely HMMs outperform PSSMs, however as the reviewer says, that is a topic that would required a dedicated investigation. Here our goal was to used domain architectures for functional comparative genomics, and we agree that a similar approach could be implemented using PSSM.  **
****
** Regarding usability, we have used SAPP (semantic annotation platform with provenance) for genome analysis and annotation. SAPP is able to store the results in the RDF data model, that can be then queried using SPARQL. This tool is available with a web interface and is available at http://semantics.systemsbiology.nl/**
2. The comparison performed in this manuscript appears to fall prey to a straw man argument. In some cases, but not all, re-writing the relevant sections of the manuscript would help to avoid any misconceptions in this regard.
  The issue of replacing a O(n2) cost with a O(n) one upon addition of a new genome was dealt with over 15 years ago, so the statement "On the other hand, addition of a new genome using an SB approach require a new set of all-against-all sequence comparisons which come at a O(n2) computational cost" is false - at least as it is currently written.
**We have amended the above mentioned sentence to:**
****
** On the other hand, addition of a new genome using an SB approach require a new set of all-against-all sequence comparisons which come at a *O*( *n*^2^) computational cost. However, approaches has been proposed to overcome this shortcomings of SB methods, such as COGNITOR which reduces the computational to *O*( *n*) by using pre-computed databases.**
3. It is true that building groups of orthologs do require an initial O(n2) computational cost, but once those orthologous groups are formed, methods such as COGNITOR (first published in the year 2000) work extremely quickly and efficiently to assign genes in newly-sequenced genomes to existing groups. In fact, COGNITOR works in the exact same manner in which DAB uses pre-computed domain databases to achieve the much lower O(n) cost, although in COGNITOR's case it searches against a pre-computed database of orthologous groups (of which there are far fewer than domains, so with a smaller "n" it would actually be faster than DAB).
  In should be noted that despite DAB's somewhat higher cost, it has the theoretical potential to achieve better accuracy than COGNITOR (at least in some cases) since as a global sequence similarity approach, the latter does not explicitly consider domain architecture. At least not in an automated fashion - doing so would require manual curation of its results, which is often done by careful researchers, but is not a process that is scalable to handle the ever-decreasing cost and ever-increasing amounts of genomic data.
**We have commented on the analogy between DAB and COGNITOR.**
****
** In this respect, the DAB approach is similar in to the approach implemented in COGNITOR, by searching against existing databases of domains architectures.**
4. Although since a comparison with COGNITOR was not included in the manuscript, either in terms of speed or accuracy, it is unknown how much more useful DAB would be in practice.
**The focus of the paper was not to propose a comparative analysis of different methods but rather to present and contextualize the use of domain architecture for comparative genomics. However, we want to stress that we are not claiming that DA methods are superior to SB but that are an efficient and scalable alternative.**
5. Even the initial O(n2) cost does not have to be terribly burdensome, since the SIMAP method pre-computes and stores BLAST results between all pairs of sequenced genomes anyway, and then uses efficient database retrieval methods to report the stored results. When a new genome is added, O(n) new comparisons have to be made - for a total accumulated cost of O(n2), although with the work spread out over many years - and these in turn are useful for many other purposes, thus mitigating the construction costs. For instance, the EGGNOG database uses this method to build groups of orthologs.
  Why was only a single SB method chosen to be a representative for this entire class of approaches? Multiple forms of DAB were tested, whereas the only SB method used for comparison was one that uses a strict e-value cutoff of 1e-5, in the form of OrthaGogue and the OrthoMCL method. Also, why was the latter chosen to be this single representative?
**We have added the following to the discussion:**
****
** To asses whether DAB results were consistent with those of SB methods we chosen. OrthaGogue as a representative of the latter class. Several tools such as COGNITOR and MultiPARANOID are available that implement different algorithm solutions to the task of identifying homologous sequences;  however, despite different implementation, they all rely on sequence similarity as a proxy for functional equivalence. Here we considered SB methods as a golden standard for functional comparative genomics, especially when organisms within close evolutionary proximity are considered. Our aim was to investigate whether using HMMs instead of sequence similarity would yield similar results, thereby justifying their use for large scale functional genome comparisons. Regarding domain architectures, we have explored different alternatives, as we have seen that the chosen database or set of reference domains plays a critical role, an example is the low coverage of TIGRFAM preventing obtention of reasonable clusters.**
6. The latter approach was designed (nearly a decade and a half ago) for eukaryotic organisms, and while it has been applied more recently to bacteria as well, it is by no means the only - or even necessarily the best - approach for prokaryotic genomes.
  One advantage that it has is that it is completely automated, and thus is "easy" for people to use (even if, as this manuscript points out, horribly slow due to the O(n2) procedure that it uses).
  On the other hand, methods like CDART and COGTRIANGLES are all also automated (the latter of which uses no arbitrary e-value cutoff - that is, the results are robust to e-values over an immense range such as 1e-5, 1, 10, or even well beyond that on up to 100, or even 1000), and some pre-computed databases (such as COGs, representing the protein families present in the last common ancestor of all cellular life several billions of years ago) even take advantage of further manual validation, and from which pre-computed groups can be identified in newly-sequenced genomes by the fully automated and even easier approaches such as DELTA-BLAST and COGNITOR. Is it at least possible that the poorer performance of SB methods in comparison to DAB as shown in the current manuscript is due to the choice of this particular SB method? I for one would have loved to see a comparison against the new release of the COGs database last year, since due to its being manually curated it acts as a sort of "Gold Standard" that can be tested against, with perhaps the EGGNOG groups being used as a more realistic measure of what a purely automated method can do without human supervision. Likely, DAB would fall somewhere in-between, and which would benefit the community of researchers who want to do comparative genomics of prokaryotic organisms to have a fully automated method that was demonstrated to surpass the existing fully automated methods. As it now stands though, DAB has only been shown to surpass OrthoMCL, which is not hard to do at all. Indeed, as seventh paragraph of the Discussion section (starting "Two of the most prominent...") states, unlike DAB, the SB methods were not able to cluster together the proteins with functional similarity but little sequence identity, especially across wider taxonomic ranges - which of course is what would be expected from a SB method that uses an e-value cutoff of 1e-5.
  Above and beyond the choice of SB method, it also seems that there may have been a bug in its implementation. The statement "For SB clustering we also observed the case of identical protein sequences not clustered together, probably because of the tie breaking implementation when BBH are scored." However, this was not supposed to happen, due to the within-species reciprocal BBH procedure that is used. In contrast, the tie breaking refers to between-species comparisons, but as shown in Figure 1 of the OrthoMCL paper (http://www.ncbi.nlm.nih.gov/pubmed/12952885), these two sources of information were supposed to have been combined together to form the final orthologous groups. If the proteins were highly similar (e.g., 99%) then perhaps a tie-breaking could be explained, but for 100% identical proteins - e.g., produced by a tanden duplication event - then they should have been collected into the group. One possibility is that this particular SB method simply was not designed to handle the large numbers of extremely closely-related genome assemblies that are available today, since at the time, very few instances of multiple genomic assemblies were available for the same species. If this explanation was demonstrated to be the reason why these identical proteins were not clustered together, that would be another reason for a user to choose to use DAB over this particular SB method. In any case (bug, design flaw, or something else), this event could greatly contribute to explaining some of the results that were observed whereby this single SB method found so many more singletons than DAB with Pfam - i.e., fixing the bug, or using some other SB method, may move many of those singletons into clusters. Although it would not explain why DAB with InterPro found even more singletons than this SB method?
**We have added a paragraph in the discussion regarding why  the InterPro hierarchy has to be taken into account, also we mention this in the conclusion section.  The hierarchical structure  produces an increase in the domain multiplicity as many are related to each other. As a results an artificial variability in the DA is introduced leading to a higher number of singletons.  **
7. DAB has a lot of potential, but its limitations need to be made more clear.
**We have added a new section to the Discussion: Limitations of DAB approaches**
8. Why and how is the matrix of domain architecture binarized? Specifically, what if multiple copies of a domain are present?
**We understand that our phrasing may have caused some confusion and we apologize for unclarity. The matrix of domain architectures is only binarized (presence/absence) to compute the PCA shown in Fig. 8, not  to compare DAB and SB clustering.**
****
** We have rephrased this in the Materials and Methods section:**
** ...a binarized presence-absence matrix was obtained and used solely for principal component analysis.**
9. [..] does order matter - such as the architectures shown in Figure 2 of "A+B" and "B+A"? So, would "B+A+A" be a different architecture? And, as another reviewer also pointed out, what about "complicated" domain topologies where domains are interrupted by the insertion of another domain? Another major aspect of partial topologies is if DAB only recognizes some but not all of a newly-discovered architecture. E.g., a protein with architecture A+B+C+D, where A is known but B, C and D domains are not yet known. How would this be handled by DAB? Would it be reduced to appear merely as a single-domain "A" architecture? If so, how could that be distinguished from an architecture such as A+Z, which would also be reduced to appear just as a single-domain A? It seems like global sequence similarity methods might be more useful in those particular scenarios? i.e., if all the above domains were the same length, and a coverage threshold was used, then A+B+C+D could not be put into the same group as A+Z and A. Therefore, DAB seems primarily useful to either quickly extend known architectural types into a newly sequenced genome, but at the cost of not being able to work with unknown types.
  For newly sequenced genomes that are not yet well-characterized enough to have all of their domains present in the domain databases, DAB can be severely handicapped in comparison to global sequence similarity methods that do not have this limitation. In particular, Table 1 shows that up to nearly a fifth of the H. pylori and Cornebacteriales genomes are not able to be assigned to domain families. Even these numbers are merely lower-bound estimates, since brand-new architectures are expected to be discovered constantly, and yet these may incorporate at least one element that is known - such as the aforementioned A+B+C+D architecture, where only the A domain is represented in Pfam, but B and C and D are unknown. And yet it seems likely that even the fact that these domains are unknown would go unrecognized by the DAB approach - unless a factor is added to look for large segments of a gene that do not have matches in the databases of known domains. Therefore, the cost of DAB not being able to work with unknown architectural types might be quite high indeed. Worse, the exact value of that cost is also likewise unknown, and yet it would seem to be the single crucial piece of information that is most sorely needed in order to answer the question: does the benefits of DAB outweigh its costs?
**The reviewer raises a very interesting point regarding how extensive available knowledge on protein domains is. The high agreement between the results of DAB and SB methods is only possible because databases of protein domains have enough information. Still, we believe many domains remain to be identified and in the scenarios the reviewer mentions DAB methods will be limited. We have added the following to the Discussion section, under the “Limitations of DAB approaches” header.**
****
** Still around 15% of the genome coding content corresponds to sequences with no identified protein domains. DAB approaches can be complemented with SB methods to consider these sequences or even protein sequences with low domain coverage, possible indicating the location of protein domains yet to be identified.  **
**We have extended the paragraph in the Materials and Methods where domain architectures are defined to further emphasize that  N- C- terminal domain order is an inherent part of domain architecture definition.  **
****
** Labels indicating N-C terminal order of identified domains were assigned to each protein using the starting position of the domains: the same labels were assigned to proteins sharing the same domain architecture.**
****
** In the Introduction we have added a paragraph regarding the use of protein domain architecture in protein annotations and we have included references to previous works showing that domain order is often key for the function of the protein and that domain duplications/insertions can also alter the function of the protein.**
****
** Moreover, a similar point on how domain architectures were defined and how the hierarchical relationships between protein domains, families and clans has been raised by R. Finn and a paragraph has been added in the Discussion (see answer to R. Finn’s comments).**
10. If the goal is to bring together groups of proteins that have functional equivalence, then why was the only comparison that was done performed against the presence/absence membership of SB orthology approaches? Would it not have been better to actually measure the functional consistency observed within the SB groups, and within the DAB groups, in order to show that the latter was higher than the former? Many other methods that purport to improve upon the state-of-the-art orthology prediction process do just that - for instance Figure 4 of http://www.ncbi.nlm.nih.gov/pubmed/19148271 shows several comparisons with similarity of GO terms, enzyme nomenclature (EC), gene expression, and syntenic local neighborhood tests, with 12 different methods of orthology prediction. While neighborhood conservation is irrelevant for the issue of functional equivalence, the former three (or at least GO terms) would help to answer whether DAB is truly better than SB at the task of measuring functional equivalence. It would also help to answer whether this improved functional equivalence would be outweighed by the costs of being unable to handle unknown domain architectures, especially for highly divergent new genomes. If not, DAB may still be useful to check the consistency of existing orthologous groups in terms of their architecture, at least when domain architectures are expected to be completely known in advance - e.g., microevolutionary variations within a species where mutational events may disrupt a protein's function - but for other tasks such as the discovery of a new phyla of cellular life that contains radically different domain architectures, global similarity methods may be preferable instead.
**We have added the following section dedicated to limitations of DAB methods:**
**We have shown that domain architecture-based methods can be used as an effective approach to identify clusters of functionally equivalent proteins, leading to results similar to those obtained by classical methods based on sequence similarity. However, whether DAB methods are more accurate than SB methods to assess functional equivalence will require further analysis. In this light, results of functional conservation for both approaches could be compared in terms of GO similarity and/or EC number.**
****
** The performance of DAB methods may be sub-optimal when dealing with newly sequenced genomes that are not yet well-characterized enough to have all of their domains present in domain databases, since DAB methods will be unable to handle unknown architectural types.**
**Around 15% of the genome coding content corresponds to sequences with no identified protein domains. DAB approaches can be complemented with SB methods to consider these sequences or even protein sequences with low domain coverage, possible indicating the location of protein domains yet to be identified. Since DAB methods rely on the constant upgrading of public resources like UniProt and Pfam databases, an initial assessment of domain coverage appears as a sine qua non condition for application of these methods.**
**DAB approaches could be used to assess the consistency of existing orthologous groups in terms of their domain architectures, at least when domain architectures are expected to be completely known in advance (for instance in the case of micro-evolutionary variations within a species where mutational events may disrupt a protein's function). For other purposes, such as the discovery of a new phyla of cellular life that contains radically different domain architectures, global similarity methods may be preferred.**
11. Finally, some minor points concerning Figure 2:
  The vertical arrows seem to be pointing the wrong direction - a gene sequence undoubtedly contains more information content than a mere functional description. e.g., if I were to give you a GO code for molecular function, or biological process, then I could not tell you whether the original gene sequence is closer to one type of bacteria vs another type; but if I had the original gene sequence, then I could answer that question as well as many more.
  I did not see a description of how amino acid coordinates are used anywhere else in the manuscript, either in DAB itself or in the comparison? In short, what does "Structure" have to do with anything, other than the general theoretical flow of "sequence begets structure which begets function"? If the purpose of Figure 2 is to describe the flowchart of DAB specifically though, it should focus only on the relevant elements. I suppose Structure could have meant how the sequence alignment was made, but if that were true, then DAB would only work for domain families for which a structure is available, instead of those for which only genomic or individual gene sequence has been provided.
  The ordering also seems unclear - wouldn't BBHs inform HMM domains, which then in turn inform domain architectures? Or if starting with BBHs, then how could architectures possibly be known prior to knowing the domains themselves? Or if it should be read from top to bottom as shown, how exactly does one start with Function (e.g., a GO term) and then, somehow via Structure, thereby arrive at a Sequence alignment? Specifically, is a Pfam entry a "Function", from which the Sequence alignment is downloaded? Or are Function and the Sequence alignment both part of the starting Pfam entry (and then again, what does any of that have to do with Structure)? From which domains are found (but aren't Pfam entries domains to begin with?), and then BBHs are made from the domain architectures? (an extremely different way of doing the BBH procedure, which is normally done via Sequence alignments). In any case, as pointed out by other reviewers, the methodology used by DAB is not clearly explained in this figure, nor in the manuscript text.
**We have edited the Figure for clarity incorporating the reviewer’s suggestions.**
12. Also, the last paragraph of the Discussion uses the word "closeness", but I think "closedness" was intended.
**The typo has been amended.**

# Peer review: Referee response for version 1

**DOI:** 10.5256/f1000research.10140.r15680

**Published:** 2016-09-06

**Authors:** Robert Finn

**Affiliations:** 1 European Molecular Biology Laboratory, European Bioinformatics Institute, Cambridge, UK

The article by Koehorst *et al.* describes a comparison of two approaches for clustering genomes sequences for the purpose of performing comparative genomics. The principle behind the two approaches, sequenced based clustering and domain based clustering, is described well in the introduction. The motivation of the article is clear and well founded. However, the details provided about how domain assignments are actually performed and handled throughout the experiment generated so many questions, these have clouded the validity of any conclusions. 

  

1. How was InterPro used to assign a domain architecture? As the database presents a hierarchy of protein families and domains, unlike Pfam and TIGRFAM, there are numerous overlaps between the entries. Some of these are trivial C-terminal to N-terminal overlaps, while others are complex arrangements that cannot be simply represented as described. If three overlapping domains from InterPro are in the same hierarchy, which domain is used? If all member databases are used, this will account for the explosion of clusters in the InterPro based-clustering seen in Table 1. If InterPro accessions are used (e.g. as seen in the condensed view of a sequence on the InterPro website) then numbers are surprising.
2. How were Family vs Domain “types” handled from InterPro or Pfam? In InterPro, type families tend to be near full length protein families. In Pfam, they represent a more heterogeneous bag of entries that are yet to be established as a ‘domain’.
3. Pfam has a notion of related families, termed clans. Here the entries may not be intended to represent functionally distinct domains, but rather can represent a collection of families representing a continuum of evolution. How are entries belonging to a clan handled? How would the results differ if entries in one clan were treated as a single entity, for example, all P-loop NTPases as CL0023?  How does this influence the sequence cluster to domain architecture relationships (schematicly shown in Figure 5).
4. Why was the N-terminal starting position used to assess position of the domain? What is the effect of choosing the mid-point?
5. Both Pfam and TIGRFAM use HMMER version 3, which uses local-local alignment algorithm. How are partial hits to an HMM handled? Would two partial domain matches that occur due to an insertion between two halves of a domain be treated differently (see Triant and Pearson, 2015)? 

 

 Other comments:

1. The use of domain architectures as an approach for accelerating sequence searching is not that novel, for example, CD-ART has been available for many years. Domain architecture views have been present in most domain databases (e.g. Pfam, SMART, Prosite) for over a decade, and used in genomic contexts. A more extensive overview of the use of domain architectures in the field is desirable.
2. The composite graphs presented in Figures 6, 7 and supplementary figures use different scales, so make the graphs hard to compare.
3. When the domain based clusters are compared to the sequence based clusters, it would be interesting to understand whether the number of domains that makes up the domain architecture influences the correlations to the sequence based clusters. Do single domain architectures predominated the 1:1 clusters?
4. Many readers may be unaware of the thresholds employed in InterProScan relate to the individual databases, so greater clarity is required.
5. Why is the versioned InterProScan described as a semantic wrapper? 

I have read this submission. I believe that I have an appropriate level of expertise to confirm that it is of an acceptable scientific standard, however I have significant reservations, as outlined above.

## References

[1] TriantDAPearsonWR: Most partial domains in proteins are alignment and annotation artifacts. *Genome Biol*.2015;16: 10.1186/s13059-015-0656-7 99 10.1186/s13059-015-0656-7 25976240PMC4443539

[2] GeerLYDomrachevMLipmanDJBryantSH: CDART: protein homology by domain architecture. *Genome Res*.2002;12(10) : 10.1101/gr.278202 1619-23 10.1101/gr.278202 12368255PMC187533

# Response

**Published:** 2016-11-09

**Authors:** Jasper Koehorst

**Thank you for the review, we have responded to your comments below:**

1. How was InterPro used to assign a domain architecture? As the database presents a hierarchy of protein families and domains, unlike Pfam and TIGRFAM, there are numerous overlaps between the entries. Some of these are trivial C-terminal to N-terminal overlaps, while others are complex arrangements that cannot be simply represented as described. If three overlapping domains from InterPro are in the same hierarchy, which domain is used? If all member databases are used, this will account for the explosion of clusters in the InterPro based-clustering seen in Table 1. If InterPro accessions are used (e.g. as seen in the condensed view of a sequence on the InterPro website) then numbers are surprising.
**All member databases in InterPro were used. We partly took into account trivial N- terminal overlaps by alphabetically ordering the domains when distances between starting position were <3 amino acids. After analysing the results, we agree that this was not enough and this is the most likely cause of the explosion of this of clusters. As the reviewer suggests, taking the the full hierarchy of protein families and domains within InterPro would be required for comparative genome analysis based on domain architectures.**
****
** We have now better explained the selection criteria in the Materials and Methods section:**
****
** The positions (start and end on the protein sequence) of domains having Pfam, TIGRFAMs and InterPro identifiers were extracted through SPARQL querying of the graph database and domain architectures were retrieved for each protein individually. InterPro aggregates protein domain signatures from different databases. Here no pruning for redundancies has been done. Identification of domains was done using the intrinsic InterPro cut-off that represents in each case the e-values and the scoring systems of the member databases. The domain starting position was used to assess relative position in the case of overlapping domains; alphabetic ordering was used to order domains with the same starting position or when the distance between the starting position of overlapping domains was <3 amino acids. Labels indicating N-C terminal order of identified domains were assigned to each protein in such a way that the same labels were assigned to proteins sharing the same domain architecture.**
** We have commented on this point  in the discussion, where the use of InterPro is addressed.**
****
** This paragraph now reads:**
**     **
** The chosen set of domain models and the database used as a reference greatly impact the results. InterPro aggregates protein domain signatures from different databases, which leads to redundancy of the domain models. This redundancy causes overlaps between the entries and an increase of the granularity of the clusters retrieved: this can bias downwards the size of the pan-genome and upwards the size of the core- genome, as shown in Table 1. In InterPro this redundancy is taken into account by implementing a hierarchy of protein families and domains. The entries at the top of these hierarchies correspond to broad families or domains that share higher level structure and/or function; the entries at the bottom correspond to specific functional subfamilies or structural/functional subclasses of domains \cite{mitchell_interpro_2015}. Using InterPro for DAB clustering would require taking into account the hierarchy of protein families and domains: however, this would pose challenges of its own and would require discrimination of the functional equivalence of different signatures within the same hierarchy.**
****
** We have also added the following to the conclusion**
** To enable DAB approaches for highly structured databases, such as InterPro, the hierarchy of protein families and domains within has to be explicitly considered.**
2. How were Family vs Domain “types” handled from InterPro or Pfam? In InterPro, type families tend to be near full length protein families. In Pfam, they represent a more heterogeneous bag of entries that are yet to be established as a ‘domain’.
**No distinction has been introduced as there don’t seem to be general rules that apply to all cases. In the discussion section a paragraph has been added on the effects of the structure of the databases.**
3. Pfam has a notion of related families, termed clans. Here the entries may not be intended to represent functionally distinct domains, but rather can represent a collection of families representing a continuum of evolution. How are entries belonging to a clan handled? How would the results differ if entries in one clan were treated as a single entity, for example, all P-loop NTPases as CL0023?  How does this influence the sequence cluster to domain architecture relationships (schematicly shown in Figure 5).
**The reviewer raises here an interesting point that we have now discussed. The following has been added to the first paragraph of the discussion section.**
****
** Another source of redundancy are functionally equivalent domains from distantly related sequences. Pfam represents this notion through related families, termed clans, where relationship may be defined by similarity of sequence, structure or profile-HMM. Clans might contain functionally equivalent domains, however it is not clear whether this is always the case as the criteria for clan definition includes functional similarity but not functional equivalence. Members of a clan have diverging sequences and very often SB approaches would recognize the evolutionary distance between the sequences and group them in different clusters. If we were to assume that members of a clan are functionally equivalent and collect them in the same DA cluster, we will have a higher number of cases where a single DA cluster is split in multiple sequence clusters 1d→Ns. Also there would be higher number of cases of sequence clusters with the same DA but no exactly matching the DA clusters (1s→1d cases).**
4. Why was the N-terminal starting position used to assess position of the domain?
**The following line has been rewritten in the Methods section**
****
** Labels indicating N-C terminal order of identified domains were assigned to each protein using the starting position of the domains: the same labels were assigned to proteins sharing the same domain architecture.**
5. What is the effect of choosing the mid-point?
**We have commented on this in Results and Discussion. The following paragraph has been added:**
****
** The starting position of the domains was used to generate labels indicating N-C terminal order of identified domains. The labels were used only for clustering as proteins sharing the same labels were assigned to the same clusters. Choosing instead the mid-point or the C-terminal position could affect the labeling but it not the obtained clusters.**
6. Both Pfam and TIGRFAM use HMMER version 3, which uses local-local alignment algorithm. How are partial hits to an HMM handled? Would two partial domain matches that occur due to an insertion between two halves of a domain be treated differently (see Triant and Pearson, 2015)?
**In the discussion we have added a subsection on the limitations on DAB approaches. There we have added the following:**
****
** Partial domain hits might arise as a result of alignment, annotation and sequence assembly artifacts (cite Triant *et al.*). To reduce the number of partial domain hits additional pruning could be implemented to distinguish these cases. However, this is an open problem that requires caution as it could influence the functional capacity of an organism and clustering approaches using DA.**
7. The use of domain architectures as an approach for accelerating sequence searching is not that novel, for example, CD-ART has been available for many years. Domain architecture views have been present in most domain databases (e.g. Pfam, SMART, Prosite) for over a decade, and used in genomic contexts. A more extensive overview of the use of domain architectures in the field is desirable.
**We have added the paragraph in the introduction regarding domain architectures, comparison of domain architectures and their use for sequence search. We have also discussed on how these have been included in domain databases and, as also suggested by the first reviewer,  on the preservation of domain architectures at high phylogenetic distances.**
****
** The following paragraph has been added to the introduction:  **
****
** Domain architectures have been shown to be preserved at large phylogenetic distances both in prokaryotes and eukaryotes (Koonin 2002, Kummerfeld 2009). This lead to the use of protein domain architectures to classify and identify evolutionarily related proteins and to detect homologs even across evolutionarily distant species (Bjorklund 2005, Fong 2007, Song 2007, Lee 2009). Structural information encoded in domain architectures has also been deployed to accelerate sequence search methods and to provide better homology detection. Examples are CDART (Geer 2002) which finds homologous proteins across significant evolutionary distances using domain profiles rather than direct sequence similarity, or DeltaBlast (Boratyn 2012) where a database of pre-constructed position-specific score matrix is queried before searching a protein-sequence database. Considering protein domain content, order, recurrence and position has been shown to increase the accuracy of protein function prediction (Messih 2012) and has led to the development of tools for protein functional annotation, such as UniProt-DAAC (DougaFn 2016) which uses domain architecture comparison and classification for the automatic functional annotation of large protein sets. The systematic assessment and use of domain architectures is enabled by databases containing protein domain information such as UniProt (Uniprot Consortium 2015), Pfam (Finn 2016), TIGRFAMs (Haft 2003) and InterPro (Mitchell 2015), SMART (Letunic 2015) and PROSITE (Sigrist 2012), that also provide graphical view of domain architectures.**
8. The composite graphs presented in Figures 6, 7 and supplementary figures use different scales, so make the graphs hard to compare.  
**Figures 6 and 7 have been combined (also supplementary figures).**
9. When the domain based clusters are compared to the sequence based clusters, it would be interesting to understand whether the number of domains that makes up the domain architecture influences the correlations to the sequence based clusters.  Do single domain architectures predominated the 1:1 clusters?
**We have looked into this and single domain architectures predominated the 1:1 clusters. A table has been added to the text (Table 3).**
10. Many readers may be unaware of the thresholds employed in InterProScan relate to the individual databases, so greater clarity is required.
**This point was also raised by A. Rosato. We have further explained the selected thresholds in the material and methods.**
** Identification of domains was done using the intrinsic InterPro cut-off that represents in each case the e-values and the scoring systems of the member databases.**
11. Why is the versioned InterProScan described as a semantic wrapper? 
**This line has been re-written, now it is explained that the versioned InterProScan stores the output in the RDF data model.**

# Peer review: Referee response for version 1

**DOI:** 10.5256/f1000research.10140.r15679

**Published:** 2016-09-01

**Authors:** Antonio Rosato

**Affiliations:** 1 Department of Chemistry "Ugo Schiff", University of Florence, Sesto Fiorentino, Italy

The authors present a very detailed and insightful analysis of the performance of alignment-based vs domain-based methods for comparative genomics. For the two methods, the proteins encoded by a selection of genomes are clustered based on pairwise sequence alignments or on their domain architectures, respectively. The first method is in principle more accurate and has higher coverage, whereas the second method is significantly faster and thus more suitable to cope with the explosion of genome information.

 The authors demonstrate that domain-based methods provide results that are well in line with alignment-based methods. Consequently, their speed advantage does not compromise accuracy. In addition, the authors suggest that the Pfam database works better than InterPro for the present clustering purpose.

 This article can benefit from some improvements:

- It is not clear to me why the labels within the colored boxes representing domains of Figure 1 differ in the top panel (Domain architectures) and the bottom panel (Domains)
- The new genome annotations generated by the authors should be made available to allow others to reproduce their calculations. It would be useful to have some data on the overall difference with respect to the original annotation
- There are no details on the parameters used for domain identification such as E-value cut-offs. The latter has a strong impact on the number of singletons (1). It would be even more useful if the authors provided VMs with the complete setup for the entire procedure (from reannotation to clustering)
- The header SB is misaligned in Table 1. Why did the authors report the fraction of proteins containing at least one InterPro domain when the rest of the analysis is based on Pfam domains?
- I find the section "Comparison of DAB and SB clusters" difficult to read. In part this is due to the fact that the authors in the text describe actual numbers while Figures 6 and 7 report percentages. In particular, why should the " horizontal acquisition of the gene " reduce the sequence similarity score (i.e. increase the E-value of the blastp alignment)? Furthermore, preservation of domain architecture at high phylogenetic distances has been extensively analyzed in the literature. References should be added
- It could be useful to combine Figures 6 and 7 to have a synoptic view
- Table 1 shows that InterPro domains provide pangenomes that are not only always larger than the pangenomes obtained from Pfam domains but sometimes even larger than SB-derived pangenomes (e.g. *H. pylori *or Cyanobacteria). How is this possible?
- The low value of alpha in the Heaps regression for *L. monocytogenes* afforded by the DAB is striking and should be analyzed in more detail
- The line break after "transfer events" in the second paragraph of the introduction is not needed
- In the Supplementary material, SSB should SB

I have read this submission. I believe that I have an appropriate level of expertise to confirm that it is of an acceptable scientific standard.

## References

[1] SnipenLGUsseryDW: A domain sequence approach to pangenomics: applications to Escherichia coli. *F1000Res*.2012;1: 10.12688/f1000research.1-19.v2 19 10.12688/f1000research.1-19.v2 24555018PMC3901455

# Response

**Published:** 2016-11-09

**Authors:** Jasper Koehorst

1. It is not clear to me why the labels within the colored boxes representing domains of Figure 1 differ in the top panel (Domain architectures) and the bottom panel (Domains)
**In the older version the labels in the top referred to the domain names whereas the labels on the bottom contained the PFAM identifiers. The figure has been changed so that only one set of labels is presented.**
2. The new genome annotations generated by the authors should be made available to allow others to reproduce their calculations. It would be useful to have some data on the overall difference with respect to the original annotation.
**The reviewer raises a very interesting topic that has been the focus of a different study. We have performed a detailed analysis of the differences between the original and the *de novo *annotation in a set of 432 Pseudomonas genomes. In that case, an average difference of 153 genes per genome was detected. Differences in annotations were observed at all functional levels (EC numbers, GO terms and protein domains). The magnitude of the differences correlated with the date the original annotation. The manuscript is currently under review and we will include the reference as soon as it is published.**
****
** The SAPP annotation framework used to generate these files can be found at http://semantics.systemsbiology.nl/. Extensive documentation is available at http://sapp.readthedocs.io. A section (reproducibility) has been added indicating the workflow to reproduce the analysis here presented. We have included how annotations are compared.**
3. There are no details on the parameters used for domain identification such as E-value cut-offs. The latter has a strong impact on the number of singletons (1).
**We agree that the choice of the E-value cut off plays a critical role on domain detection and greatly impacts the size of the core-genome. However, as reported in InterPro: “The signatures contained within InterPro are produced in different ways by different member databases, so their E-values and/or scoring systems cannot be meaningfully compared” therefore we have selected the intrinsic cutoff within InterPro [Mitchel *et al* 2015]. This has been mentioned in the Material and Methods section:**
****
** Identification of domains was done using the intrinsic InterPro cut-off that represents in each case the E-values and the scoring systems of the member databases (Mitchel 2015).**
4. It would be even more useful if the authors provided VMs with the complete setup for the entire procedure (from reannotation to clustering)
**The SAPP annotation framework used to generate these files can be found at http://semantics.systemsbiology.nl/. Extensive documentation is available at:  http://sapp.readthedocs.io.  A section has been added indicating the workflow to reproduce the analysis here presented.**
5. The header SB is misaligned in Table 1. Why did the authors report the fraction of proteins containing at least one InterPro domain when the rest of the analysis is based on Pfam domains?
**We have modified Table 1 and included an additional column with the fraction of proteins containing at least one Pfam domain.**
6. I find the section "Comparison of DAB and SB clusters" difficult to read. In part this is due to the fact that the authors in the text describe actual numbers while Figures 6 and 7 report percentages. In particular, why should the " horizontal acquisition of the gene " reduce the sequence similarity score (i.e. increase the E-value of the blastp alignment)?
**We have rephrased the sentence on horizontal gene acquisition, it now reads:**
** Similarly, there are 399 1s → 1d clusters. Each of these cases represent a sequence cluster where all the sequences share the same domain architecture, but other sequences exist with the same architecture that have not been included in the cluster due to a too low similarity score. The low similarity between sequences with the same domain architecture could be due to  a horizontal acquisition of the gene  or to a fast protein evolution at the sequence level. Genes acquired from high phylogenetic distances could greatly vary in sequence while presenting the same domain architecture.  **
7. Furthermore, preservation of domain architecture at high phylogenetic distances has been extensively analyzed in the literature. References should be added.
**The following paragraph has been added to the introduction:  **
** Domain architectures have been shown to be preserved at large phylogenetic distances both in prokaryotes and eukaryotes (Koonin 2002, Kummerfeld 2009). This lead to the use of protein domain architectures to classify and identify evolutionarily related proteins and to detect homologs even across evolutionarily distant species (Bjorklund 2005, Fong 2007, Song 2007, Lee 2009). Structural information encoded in domain architectures has also been deployed to accelerate sequence search methods and to provide better homology detection. Examples are CDART (Geer 2002) which finds homologous proteins across significant evolutionary distances using domain profiles rather than direct sequence similarity, or DeltaBlast (Boratyn 2012) where a database of pre-constructed a position-specific score matrix is queried before searching a protein-sequence database. Considering protein domain content, order, recurrence and position has been shown to increase the accuracy of protein function prediction (Messih 2012) and has led to the development of tools for protein functional annotation, such as UniProt-DAAC (Dougan 2016) which uses domain architecture comparison and classification for the automatic functional annotation of large protein sets. The systematic assessment and use of domain architectures is enabled by databases containing protein domain information such as UniProt (Uniprot Consortium 2015), Pfam (Finn 2016), TIGRFAMs (Haft 2003) and InterPro (Mitchell 2015), SMART (Letunic 2015)  and  PROSITE (Sigrist 2012), that also provide graphical view of domain architectures.**
8. It could be useful to combine Figures 6 and 7 to have a synoptic view
**Figures 6 and 7 (and supplementary figures) have been combined.**
9. Table 1 shows that InterPro domains provide pangenomes that are not only always larger than the pangenomes obtained from Pfam domains but sometimes even larger than SB-derived pangenomes (e.g. H. pylori or Cyanobacteria). How is this possible?    
  InterPro aggregates protein domain signatures from different databases, which leads to redundancy of the domain models. This redundancy causes overlaps between the entries and an increase of the granularity of the clusters retrieved: this can bias downwards the size of the pan-genome and upwards the size of the core- genome, as shown in Table 1.
10. The low value of alpha in the Heaps regression for L. monocytogenes afforded by the DAB is striking and should be analyzed in more detail
**We thank the reviewer for this very interesting observation. We have investigated the low value of alpha in this case and the following paragraph has been added**
****
** The alpha DAB value retrieved for *L. monocytogenes*  is strikingly low. Heaps law regression relies on the selected genomes providing a uniform sampling of selected taxon, here species. Analysis of the domain content of the selected genomes shows a divergent behaviour of strain LA111 (genome id GCA\_000382925-1). This behaviour is clear in Figure 7 (PCA), where GCA\_000382925-1 appears as an outlier of the *L.monocytogenes* group. Removal of these outlier leads to alpha DAB=1.04 and alpha SB=0.64, which emphasizes  the need for uniform sampling prior to Heaps regression analysis.**
11. The line break after "transfer events" in the second paragraph of the introduction is not needed
**The line break has been removed.**
12. In the Supplementary material, SSB should SB
**This typo has been fixed.   **
